# Molecular Mechanisms of Retinal Damage in NMOSD via Müller Glial Cell Stimulation with Patient Sera

**DOI:** 10.1007/s12035-026-05892-y

**Published:** 2026-05-04

**Authors:** Sahra Kabiri, İrfan Burak Göloğlu, Ahmetcan Sezen, Ceren Tuncer, Mohammad Haroon Qureshi, Rabia Gökçen Gözübatik Çelik, Afsun Şahin, Ayşe Altıntaş

**Affiliations:** 1 Graduate School of Health Sciences (GSHS), Koç University, Istanbul, Turkey; 2 Koç University Research Center for Translational Medicine (KUTTAM), Koç University, Istanbul, Turkey; 3 Department of Neurology, Koç University School of Medicine, Istanbul, Turkey; 4 Department of Ophthalmology, Koç University School of Medicine, Istanbul, Turkey; 5 School of Medicine, Koç University, Istanbul, Turkey; 6 Department of Molecular Biology and Genetics, Boğaziçi University, Istanbul, 34342 Turkey; 7 German Primate Center, Leibniz Institute for Primate Research, Kellnerweg 4, Göttingen, 37077 Germany; 8 Department of Neurology, Bakirkoy Prof. Dr. Mazhar Osman Training and Research Hospital for Mental Health and Neurological Disorders, University of Health Sciences, Istanbul, Turkey

**Keywords:** Neuromyelitis optica spectrum disorder, Optic neuritis, Retinal pathology, Müller cells, Serum-mediated effects, Antibody-independent pathways

## Abstract

Neuromyelitis optica spectrum disorder (NMOSD) is a rare autoimmune CNS disease that frequently causes severe optic neuritis, yet the molecular mechanisms driving retinal damage remain incompletely understood, especially across different NMOSD subgroups. Müller glial cells, which maintain retinal water–ion homeostasis through AQP4 and Kir4.1 channels, may represent a primary retinal target of circulating NMOSD-related autoantibodies and serum factors. We investigated how sera from AQP4-IgG+, MOG-IgG+, and double-seronegative (DSN) NMOSD patients affect the expression and localization of key Müller cell biomarkers. Human MIO-M1 Müller cells were stimulated with complement-inactivated patient or healthy control sera, and protein/mRNA levels of AQP4, Kir4.1, CRALBP, VEGF, and IL-6 were evaluated using immunofluorescence, Western blotting, and RT-qPCR. AQP4 membrane localization and internalization were assessed using WGA and EEA1 colocalization analyses. AQP4-IgG+ sera uniquely induced a marked reduction in AQP4 and Kir4.1 protein expression, together with mild AQP4 internalization and reduced membrane association. Despite protein loss, AQP4 and Kir4.1 transcripts were significantly upregulated, indicating a compensatory transcriptional response to antibody-mediated depletion. MOG-IgG + sera produced no major changes in the examined markers. In contrast, DSN sera selectively increased VEGF expression at both protein and mRNA levels, suggesting an alternative, antibody-independent mechanism of Müller cell activation. IL-6 expression showed non-significant changes across groups. These findings demonstrate subgroup-specific retinal glial responses to patient sera, with AQP4-IgG mediating early complement-independent loss of the AQP4–Kir4.1 water–ion channel complex, and DSN sera engaging distinct VEGF-related pathways. Our study establishes Müller cells as active contributors to NMOSD-associated retinal pathology and provides a foundation for exploring subgroup-tailored changes.

**Supplementary Information:**

The online version contains supplementary material available at 10.1007/s12035-026-05892-y.

## Introduction

Neuromyelitis optica spectrum disorder (NMOSD) is a rare, severe autoimmune disease of the central nervous system (CNS) characterized by the production of pathogenic autoantibodies against aquaporin-4 (AQP4), the primary water channel of the CNS [1, 2]. AQP4-IgG antibodies penetrate the CNS through areas of blood–brain barrier (BBB) disruption and bind to AQP4 water channels located on perivascular astrocyte endfeet at the CNS–blood interface, as well as on subpial and subependymal astrocyte processes at the CNS-cerebrospinal fluid (CSF) interface and on retinal Müller cells. This binding results in granulocyte infiltration and complement system activation, which results in astrocyte and Müller cell injury, secondary demyelination, and finally neuronal loss [3, 4].

Clinically, NMOSD most often presents with severe optic neuritis (ON) and longitudinally extensive transverse myelitis (LETM), whereas less frequent manifestations include area postrema syndrome (APS), acute brainstem or diencephalic syndromes such as symptomatic narcolepsy, and symptomatic cerebral involvement [5]. Although AQP4-IgG remains a central diagnostic biomarker, it is detectable only in 60–80% of patients with NMOSD. About 21% to 27% of seronegative patients are positive for antibodies against myelin oligodendrocyte glycoprotein (MOG) [6]. A subset of patients remains seronegative for both AQP4-IgG and MOG-IgG; these are defined as double seronegative (DSN) NMOSD, representing the least well-characterized subgroup [7].

Optic neuritis is one of the most common and debilitating manifestations, which often leads to irreversible vision loss due to retinal and optic nerve damage [8]. Retinal involvement, especially in AQP4-IgG + patients, includes subclinical thinning of the ganglion cell–inner plexiform layer (GCIPL) and the peripapillary retinal nerve fiber layer (pRNFL), even without ON symptoms, suggesting the imperative role of circulating antibody-mediated injury to astrocytes and Müller glial cells [9].

**Fig. 1:**
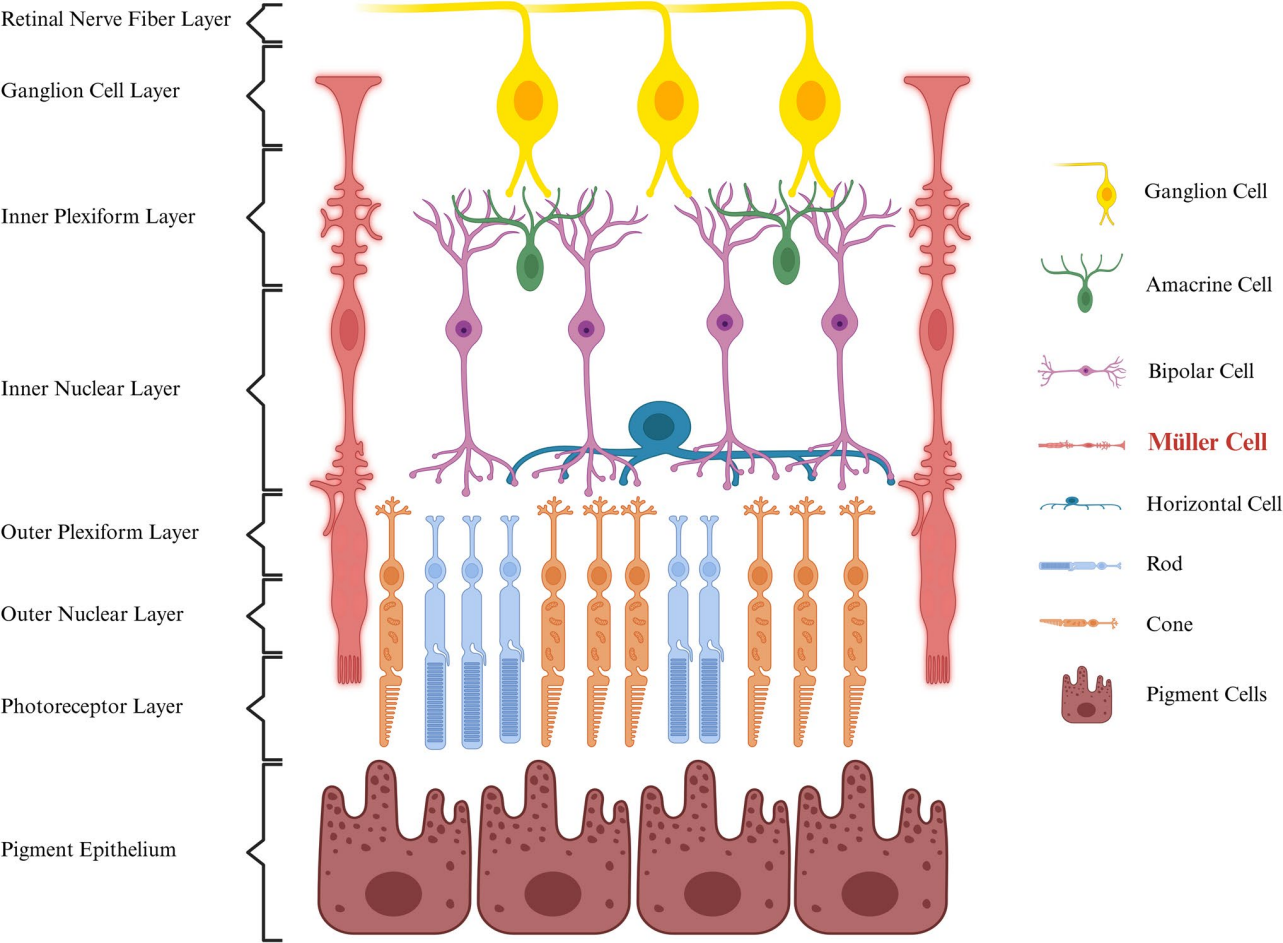
Localization of Müller cell in complex retinal cellular organization. Created with BioRender

Müller cells are the principal and most abundant glial cells in the retina, which span the entire retinal thickness and maintain close contact with retinal neurons, as shown in Fig. 1. They are crucial for retinal homeostasis, contributing to neurotransmitter recycling and ion and water regulation via Kir4.1 and AQP4 membrane proteins subsequently [10]. They also exert neuroprotective effects by scavenging free radicals and secreting neurotrophic factors and antioxidants such as glutathione. By forming the inner and outer limiting membranes and interacting with vascular endothelial cells, Müller cells contribute to the blood–retina barrier (BRB) and maintain tissue homeostasis [11]. Loss of AQP4 caused by autoantibodies in NMOSD leads to Müller cell swelling, which in turn triggers an inflammatory response and damages retinal ganglion cells and the retinal microvasculature [12, 13]. This underscores Müller cells as a critical retinal target in NMOSD pathology.

A comparative assessment of retinal effects across different NMOSD subgroups (AQP4-IgG+, MOG-IgG+, and DSN) can reveal previously unrecognized pathogenic pathways and broaden our understanding of disease heterogeneity. Despite significant progress in understanding NMOSD pathogenesis, the role of Müller cells in NMOSD-associated retinal damage remains largely unexplored. Given the crucial role of Müller glial cells in maintaining retinal homeostasis and their involvement in neuroinflammatory processes, investigating their response to disease-related factors can provide valuable insights into retinal pathology associated with NMOSD.

In this study, we aimed to elucidate molecular alterations in the retina by examining the expression levels of key biomarkers including AQP4, cellular retinaldehyde-binding protein (CRALBP), inwardly rectifying K + channel 4.1 (Kir4.1), vascular endothelial growth factor (VEGF), and interleukin-6 (IL-6) in the human Moorfields/Institute of Ophthalmology-Müller 1 (MIO-M1) Müller glial cell line [14] following stimulation with patient sera from different NMOSD subgroups. Given the rarity of NMOSD, particularly double-seronegative cases, this study was designed as an exploratory pilot investigation to characterize Müller cell responses to patient sera to identify potential subgroup-specific patterns that may warrant further investigation in larger cohorts.

## Methods and Materials

### Study Participants

NMOSD patients were recruited from the Neurology Outpatient Clinic of Koç University Hospital and fulfilled the criteria established by the 2015 International Panel for NMO Diagnosis (IPND) [1] (Table 1). This study included four sample groups: three AQP4-IgG+ NMOSD patients, three MOG-associated Disease (MOGAD) patients with NMOSD phenotype, three DSN-NMOSD patients, and three healthy-controls (HCs). All participants provided written informed consent (Koç University Institutional Review Board approved the study with the ethical number: 2022.303.IRB2.050), and the patients’ sera were confirmed for AQP4-IgG by a commercial cell-based assay and MOG-IgG positivity by a live-cell-based assay.

**Table 1:** Clinical features of the selected patient subgroups

Parameter/groups	AQP4-1	AQP4-2	AQP4-3	MOG-1	MOG-2	MOG-3	DSN-1	DSN-2	DSN-3
Age at sampling (years)	45	70	67	29	58	25	41	54	40
Age at onset (years)	39	63	63	23	57	14	22	50	39
Sex	Female	Female	Female	Female	Male	Male	Female	Male	Female
Total relapses	4	3	4	3	5	3	5	2	2
ON	3	0	0	3	5	2	2	2	1
LETM	1	3	0	0	0	1	4	0	1
Brainstem involvement	1	0	4	0	0	0	0	1	0
EDSS at sampling	2,5	5,5	2	2,5	2	2	2,5	3,5	3
Immunotherapy	Azathioprine	Azathioprine	Azathioprine	Azathioprine	Rituximab + Azathioprine	None	None	Azathioprine	Azathioprine

Data are presented as mean ± standard deviation (SD) or percentage. *ARR* annualized relapse rate, *EDSS* Expanded Disability Status Scale

Serum samples were collected during remission. Patients with a history of unexplained infections, human immunodeficiency virus (HIV) infection, type 1 diabetes, or other systemic inflammatory diseases were excluded. Healthy controls were adults without known autoimmune, autoinflammatory, or chronic systemic diseases (e.g., diabetes, cancer, hepatitis, and tuberculosis). Individuals with a recent allergic reaction, infection, or use of nonsteroidal anti-inflammatory or immunomodulatory drugs within the last four weeks were excluded.

### Sample Preparation

To isolate sera, a peripheral blood sample (10 mL) from each individual was collected into a serum-separating tube. Samples were then centrifuged at 3000 × g for 10 min at 4 °C (AllegraX-12R, Beckman-Coulter, USA). The samples were aliquoted into 1-mL cryovials and stored at − 80 °C. To eliminate the effects of the complement system and enable the investigation of complement-independent antibody-mediated effects, the serum samples were heat-inactivated at 56 °C for 30 min in a block-heater before use [15].

### MIO-M1 Cell Culture and Serum Stimulation

The human Müller glial cell line MIO-M1 was cultured in Dulbecco’s Modified Eagle’s Medium–High Glucose (Sigma-Aldrich, D6429), 10% fetal bovine serum (FBS; Biowest), and 1% penicillin–streptomycin at 37 °C with 5% CO₂. Cells were then trypsinized (Multicell, 0.25% trypsin 2.21 mM EDTA 4Na) for 5 min at 37 °C after they reached 70–80% confluency to perform downstream experiments. The MIO-M1 cells were then incubated for 12 h at 37 °C with heat-inactivated sera (diluted 1:5 in serum-free medium (SFM)). Sera from each patient were used individually, such that each serum sample represented one biological replicate in all subsequent experiments. Experiments were performed in three independent experimental sets.

To assess changes in the expression of VEGF and IL-6 in Müller cells, cells were treated with Brefeldin A (BFA; 1:1000, Sigma-Aldrich). BFA inhibits protein transport by blocking the Golgi apparatus, thereby preventing the secretion of cytokines into the extracellular medium.

### Cell Metabolic Activity Assay

MTT assay was performed to evaluate the effects of patients’ sera on the metabolic activity of MIO-M1 cells. To this end, cells were seeded in 96-well plates overnight. Cells were then stimulated with different concentrations (2%, 5%, 10%, 20%) of heat-inactivated HC and AQP4-IgG+ sera in SFM for 0, 24, 48, or 72 h at 37 °C, following serum starvation using SFM for 2 h.

At each time point, MTT solution (5 mg/mL in PBS; M6494, Invitrogen, USA) was added to each well to a final concentration of 0.5 mg/mL, followed by incubation for 2 h at 37 °C. Subsequently, 75% of the MTT solution was removed, and 100 µL of DMSO:isopropanol (1:1) was added to solubilize formazan crystals. Plates were incubated for 10 min at 37 °C and mixed by pipetting to ensure complete dissolution.

Absorbance was measured at 540 nm using a BioTek Synergy H1 microplate reader (Agilent, USA). Cell metabolic activity was expressed as a percentage relative to the control group. 

### Immunofluorescence (IF) Staining

IF staining was used to evaluate the effects of patients’ sera on the expression levels of water channel (AQP4), gliosis (Kir4.1, CRALBP), and inflammatory (IL-6, VEGF) markers. Cells were first cultured on 12 mm glass coverslips and exposed to patient and HC sera for 12 h after they reached 70–80% confluency. Cells were then washed twice with 1 × PBS and then fixed with 4% paraformaldehyde (PFA) for 15 min at room temperature. Following washing with PBS, cells were permeabilized with 0.1% Triton X-100 in PBS for 10 min, then blocked with Superblock (ScyTek Laboratories, USA) for 30 min at room temperature. Primary antibodies were applied overnight at 4 °C: anti-AQP4 (1:150; ThermoFisher, PA5-53,234), anti-Kir4.1 (1:100; Thermo Fisher, PA5-77,822), anti-CRALB (1:500; Abcam, ab154898), anti-IL-6 (1:100; Thermo Fisher, P620), and anti-VEGF (1:100; Thermo Fisher, MA1-16629). On the following day, samples were washed using PBS-T (0.05% Tween20 in PBS) for three times; cells were then incubated with Alexa Fluor 488–conjugated goat anti-mouse (A11029) or goat anti-rabbit (A11008) secondary antibodies (Invitrogen, USA) for 40 min at room temperature under light-protected conditions. After incubation with secondary antibodies, coverslips were mounted on Superfrost™Plus adhesion microscope slides (Epredia, USA) using DAPI-containing mounting medium (ab104139; Abcam, UK). Images were captured using Leica DMi8S live-cell microscopy.

### Colocalization and Internalization Assay

To investigate potential alterations in AQP4 distribution across the cell membrane and its cytoplasmic internalization following sera stimulation, IF experiments were performed to analyze AQP4 colocalization and internalization separately. For colocalization analysis, wheat germ agglutinin (WGA) was used as a plasma membrane marker. Following serum stimulation, cells were first incubated with Alexa Fluor–conjugated WGA (5 µg/ml; Thermo Fisher, W11261) for 30 min at 4 °C to label the plasma membrane. Cells were then fixed and stained using AQP4 primary antibody (1:150; Thermo Fisher, PA5-53234) and appropriate Alexa Fluor–conjugated secondary antibodies (1:200) based on the previously described IF-staining protocol. For internalization analysis, early endosome antigen-1 (EEA1) was used as a marker of early endosomes. Following serum stimulation, cells were co-stained using primary antibodies against AQP4 (1:150; Thermo Fisher, PA5-53234) and EEA1 (1:300; Thermo Fisher Invitrogen, 14–9114-82) overnight at 4 °C. Appropriate Alexa Fluor–conjugated secondary antibodies (1:200) were subsequently applied for 40 min at room temperature in the dark.

### Image Analysis

#### Protein Expression Analysis

IF images were acquired using a Leica DMi8S live-cell microscope and exported as TIFF files via LASX software (Leica, Wetzlar, Germany). Images were analyzed using Image J (NIH, Bethesda, MD, USA). For quantitative assessment, fluorescence intensities of target markers were measured under identical acquisition settings. Post-capture adjustments (contrast enhancement and background subtraction) were applied uniformly across all groups.

#### Colocalization and Internalization Analysis

Confocal images were obtained using a Leica DMi8 laser scanning confocal microscope (Leica Microsystems, Germany) equipped with LASX software. Colocalization and internalization of AQP4 were analyzed using an ImageJ JaCoP colocalization plugin. Manders’ overlap coefficient (MOC) method was used to calculate AQP4 coefficient with the plasma membrane. This coefficient measures the fraction of fluorescence signal from one channel overlapping with the signal from another, ranging from 0 (no colocalization) to 1 (complete colocalization). For colocalization analysis, MOC was used to determine the proportion of AQP4 signal overlapping with WGA, indicating its localization at the plasma membrane. For internalization analysis, the coefficient was calculated to represent the overlap between AQP4 and EEA1, indicating the fraction of AQP4 internalized into early endosomes following serum stimulation.

### Western Blotting (WB)

To validate protein expression intensities measured using IF-staining, WB analyses were performed. After serum stimulation, cells were collected and lysed in radioimmunoprecipitation assay (RIPA) buffer with 1X protease and phosphatase inhibitors. Protein concentrations were determined using a bicinchoninic acid (BCA) assay kit (Thermo Scientific, USA). Equal amounts of protein (20 µg) were mixed with 4X Laemmli sample buffer (90%) containing 10% β-mercaptoethanol, denatured at 95 °C for 10 min, and resolved on 10–12% precast SDS-PAGE gels. Proteins were transferred to polyvinylidene fluoride (PVDF) membranes, which were blocked for 1 h at room temperature in 5% skim milk in TBST. The membranes were incubated overnight at 4 °C with primary antibodies against vinculin (1:1000), AQP4 (1:1000), Kir4.1 (1:1000), IL-6 (1:1000), and VEGF (1:1000). After three washes for 10 min in TBST, membranes were incubated with HRP-conjugated goat anti-rabbit or anti-mouse IgG (1:2000) for 1 h at room temperature. Immunoreactive bands were visualized using enhanced chemiluminescence (ECL) or SuperSignal™West Femto substrate (Thermo Scientific, USA) and imaged with a Bio-Rad imaging system. Protein band intensities were quantified using Image Lab software (Bio-Rad, USA).

### Real-Time Quantitative PCR (RT-qPCR)

Firstly, to evaluate the mRNA expression levels of AQP4, Kir4.1, IL-6, and VEGF markers following serum stimulation, total RNA was isolated using the Quick-RNA™ Miniprep Plus Kit (Zymo Research, USA) according to the manufacturer’s protocol. The RNA concentration and purity were determined with a NanoDrop2000 spectrophotometer (Thermo Scientific, USA), and samples were stored at − 80 °C until use.

The iScript™cDNA Synthesis Kit (Bio-Rad, USA) was used to synthesize complementary DNA (cDNA) according to the manufacturer’s instructions and stored at − 20 °C. Gene expression of AQP4, Kir4.1, VEGF, IL-6, and the reference gene GAPDH was quantified using a LightCycler®480 system (Roche, Switzerland) with LightCycler®480 SYBR Green I Master Mix. Primer sequences are listed in Table 2. Reactions were performed in 96-well plates, with nuclease-free water serving as a negative control. After optimization, an annealing temperature of 60 °C was determined to yield the most specific and efficient amplification across all primer sets. Each reaction contained 50 ng cDNA, with primer concentrations of 0.5 µM for AQP4, Kir4.1, and VEGF and 2.5 µM for IL-6 and GAPDH. Cycle threshold (Ct) values were obtained using LightCycler® software. Expression levels of target genes were normalized to GAPDH and further normalized to SFM-treated controls. Relative mRNA expression was calculated using the Pfaffl method.

**Table 2:** Primer sequences

Primer name	5′−3′ sequence	Reference
AQP4-Forward	GGAATTTCTGGCCATGCTTA	[16]
AQP4-Reverse	AGACTTGGCGATGCTGATC	
Kir4.1-Forward	CAAGGACCTGTGGACAACCT	
Kir4.1-Reverse	GGGATTCAAGGGAGAAGAGG	
VEGF-Forward	GGGCAGAATCATCACGAAGT	[17]
VEGF-Reverse	TGGTGATGTTGGACTCCTCA	
IL-6-Forward	ACATCCTCGACGGCATCTCA	In-house prep
IL-6-Reverse	CACCAGGCAAGTCTCCTCATTG	
GAPDH-Forward	TCAAGGCTGAGAACGGGAAG	[18]
GAPDH-Reverse	CGCCCCACTTGATTTTGGAG	

### Statistical Analysis

Data are presented as mean ± standard deviation (SD) from at least three independent experiments, unless otherwise stated in figure legends. For multiple-group comparisons, one-way analysis of variance (ANOVA) followed by Tukey’s post hoc test was performed. For colocalization between 2 groups, Student’s’ *t*-test was performed. A *p*-value (*p*) < 0.05 was considered statistically significant, with significance levels denoted as *p* < 0.05, *p* < 0.01, and *p* < 0.001. All statistical analyses were conducted using GraphPad Prism version 10.1.1 (GraphPad Software, San Diego, CA, USA). To analyze the cell metabolic activity assay, experiments were performed in quadruplicate, and results were presented as mean ± standard deviation. Statistical analysis was conducted using two-way ANOVA followed by Tukey’s post-hoc test. Effect sizes for pairwise comparisons were calculated using Cohen’s d based on pooled standard deviations and interpreted according to conventional thresholds (small ≥ 0.2, medium ≥ 0.5, large ≥ 0.8).

## Results

### Clinical Features of the Selected Samples

#### Patient Sera Decreased MIO-M1 Cell Metabolic Activity in a Time- and Dose-Dependent Manner

**Fig. 2:**
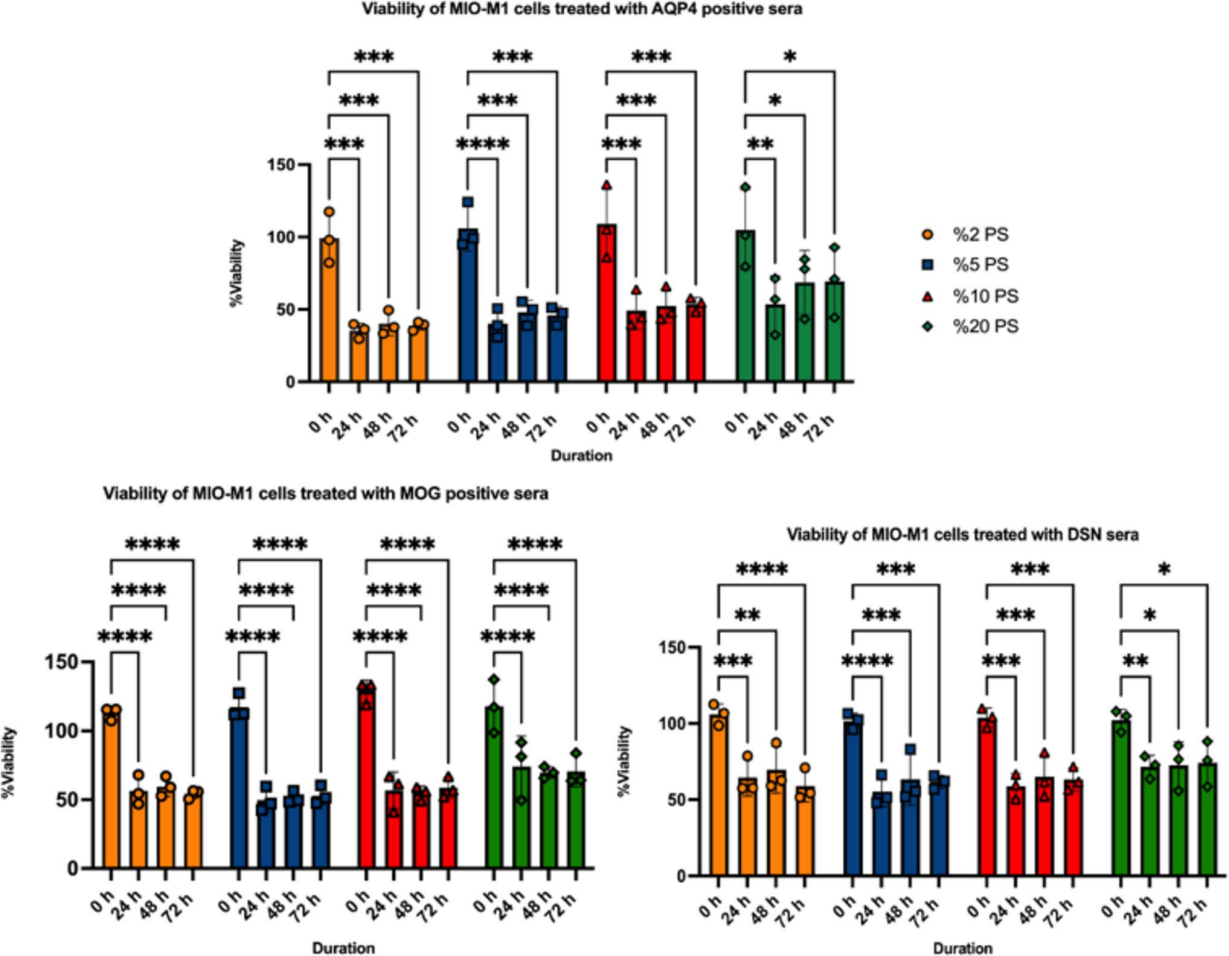
MTT assay results showing the metabolic activity of MIO-M1 cells following exposure to heat-inactivated patient sera from different subgroups at different concentrations and time points. Data are presented as mean ± standard deviation (SD). Each group included *n* = 3 independent samples (AQP4-IgG+, MOG-IgG+, and DSN). Statistical analysis was performed using two-way ANOVA. Significant differences compared with the 0 h control group are indicated as *p < 0.05, **p < 0.01, and ***p < 0.001

MTT assays revealed a significant reduction in MIO-M1 cell metabolic activity following exposure to heat-inactivated patient sera across different concentrations and time points. AQP4-IgG+ sera exhibited a distinct concentration-dependent pattern. The most pronounced reduction in metabolic activity was observed at lower serum concentrations (2%) across all time points, whereas higher concentrations (10% and 20%) resulted in comparatively attenuated effects (Fig. 2). Collectively, these findings suggest that AQP4-IgG+ sera exert a complement-independent cytotoxic effect, likely mediated by heat-stable components such as AQP4-IgG antibodies or soluble inflammatory factors.

In the MOG-IgG+ group, metabolic activity decreased markedly at 24 h compared to baseline and remained suppressed at 48 h and 72 h, with no further significant decline between later time points. A similar temporal pattern was observed in the DSN group (Fig. 2). When comparing the magnitude of effects across groups, MOG-IgG+ sera exhibited the largest reduction in metabolic activity, as reflected by greater mean differences from baseline and higher q values in Tukey’s multiple comparisons analysis. In contrast, AQP4-IgG+ and DSN sera also demonstrated significant reductions, but with comparatively smaller effect sizes under the same conditions. Notably, no significant differences were observed between later time points (24 vs. 48 h vs. 72 h) within each group, indicating that the reduction in metabolic activity occurs early and remains stable over time.

Most importantly, 20% serum more closely mimics physiological conditions and increases the likelihood of detecting the effects of a broader range of serum-derived factors, which is particularly relevant when studying subtypes such as MOG-IgG+ and DSN-NMOSD, where disease mechanisms are not dependent on AQP4 autoantibodies.

Based on our prolonged incubation of MIO-M1 cells with AQP4-IgG+ sera experiment, which resulted in AQP4 downregulation (data shown in the supplementary), and the results of our cell metabolic activity assays, a serum concentration of 20% and a prolonged incubation period of 12 h at 37 °C, reflecting physiological conditions, were selected as optimal parameters for subsequent molecular and functional analyses.

#### Protein Expression Changes in MIO-M1 Cells Confirmed by IF and WB

To evaluate the expression levels of AQP4, Kir4.1, CRALBP, VEGF, and IL-6, IF staining and WB were performed.

##### AQP4 Expression

**Fig. 3:**
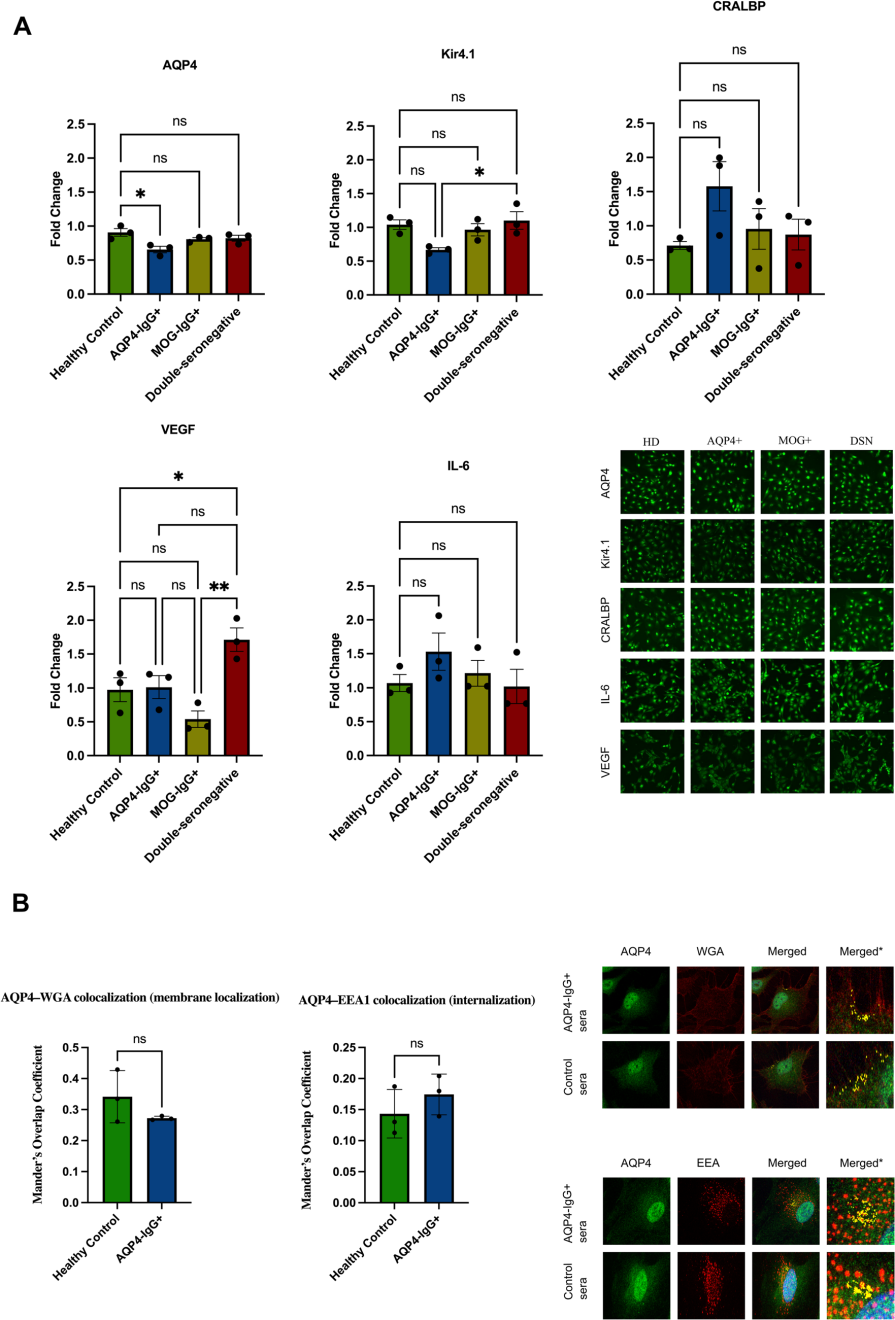
**A** IF analysis of Müller cell markers following stimulation with different NMOSD patient sera. Representative IF images and quantitative analysis of MIO-M1 cells stimulated for 12 h at 37 °C with 20% heat-inactivated sera from AQP4-IgG+, MOG-IgG+, and DSN-NMOSD patients, together with HC serum. AQP4 expression significantly decreased in cells treated with AQP4-IgG+ sera compared with HC (*p* = 0.0165), whereas MOG-IgG+ and DSN groups showed no marked difference. Kir4.1 expression was reduced in AQP4-IgG+–treated cells relative to other groups, while DSN and MOG-IgG+ conditions displayed levels comparable to HC. CRALBP staining showed a mild, non-significant increase after AQP4-IgG+ serum exposure, with minimal change in the MOG-IgG+ and DSN groups. VEGF expression was higher in DSN-treated cells compared with HC (*p* = 0.0473), whereas IL-6 levels remained largely unchanged across all experimental groups. Data are presented as mean ± SEM. Each group included *n* = 3 independent samples (AQP4-IgG+, MOG-IgG+, DSN, and HC). Statistical analysis was performed using one-way ANOVA followed by post hoc testing (*p < 0.05). **B** AQP4 localization and internalization trend in MIO-M1 cells following stimulation with AQP4-IgG + and HC sera. Representative confocal images showing AQP4 (green) and WGA (red) in MIO-M1 cells treated with either HC or AQP4-IgG + sera for 12 h at 37 °C. In AQP4-IgG+ sera–treated cells, AQP4 staining appeared less confined to the plasma membrane. Analysis of AQP4–WGA colocalization using Manders’ coefficient analysis (Fiji/ImageJ). Although a decrease in colocalization was observed after AQP4-IgG+ sera treatment, the difference was not statistically significant (*p* > 0.05). Representative confocal images showing AQP4 (green) and EEA1 (red) in MIO-M1 cells after incubation with either control or AQP4-IgG + sera for 12 h at 37 °C. Cells exposed to AQP4-IgG + sera displayed slightly enhanced intracellular punctate AQP4 signal and partial overlap with EEA1-positive vesicles. Quantification of AQP4–EEA1 colocalization using Manders’ coefficient analysis (Fiji/ImageJ). A mild, non-significant increase in colocalization was noted following AQP4-IgG + serum treatment (*p* > 0.05). Data are presented as mean ± SEM from *n* = 3 independent AQP4-IgG + sera and *n* = 3 independent healthy controls. Statistical analysis was performed using unpaired t-test followed by post hoc testing (*p < 0.05)

IF analyses revealed a reduction in AQP4 signal in MIO-M1 cells exposed to AQP4-IgG+ sera compared with those treated with HC sera (*p* = 0.0165), with a very large effect size (Cohen’s *d* = 3.27) (Fig. 3A). In contrast, cells stimulated with MOG-IgG+ or DSN sera showed no significant difference relative to the controls. These findings indicate that the decrease in AQP4 expression is specific to the presence of AQP4-IgG autoantibodies, pointing to an antibody-mediated mechanism that directly affects AQP4 expression in Müller cells.

**Fig. 4:**
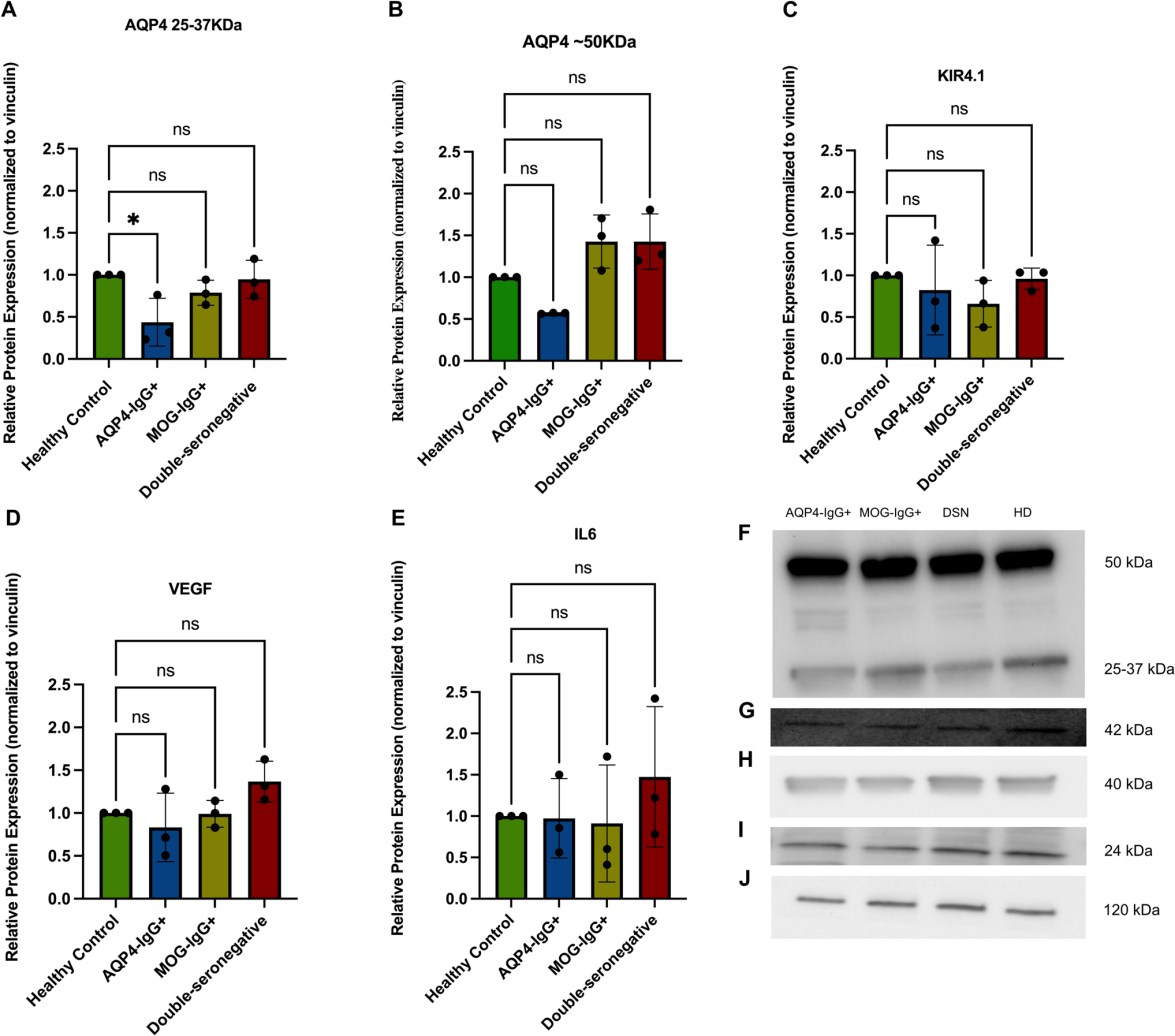
WB analysis of AQP4, Kir4.1, VEGF, and IL-6 expression in MIO-M1 cells following serum stimulation. MIO-M1 cells were stimulated for 12 h at 37 °C with 20% heat-inactivated sera from HC and NMOSD patient subgroups, including AQP4-IgG+, MOG-IgG+, and DSN cases. Representative immunoblots are shown in panels **F**–**J**. Two distinct AQP4-immunoreactive bands were consistently detected: a lower band (~ 25–37 kDa) corresponding to the M1/M23 isoforms and a higher band (~ 50 kDa) corresponding to the extended AQP4ex isoform (**F**). Kir4.1 (~ 42 kDa) (**G**), VEGF (~ 24 kDa) (**H**), and IL-6 (~ 21 kDa) (**I**) were analyzed under the same conditions, with vinculin (~ 120 kDa) used as a loading control (**J**). Quantification of relative protein expression normalized to vinculin is shown in panels **A–E**. Treatment with AQP4-IgG+ sera resulted in a significant reduction in total AQP4 (M1/M23) levels compared with HC (*p* = 0.0465), accompanied by a modest decrease in AQP4ex (B). Kir4.1 expression followed a similar downward trend in the AQP4-IgG+ group (*p* = 0.0995) (**C**). VEGF expression showed a mild, non-significant increase in DSN-treated cells (**D**), while IL-6 levels remained unchanged across all groups (**E**). Data are presented as mean ± SEM. Each group included *n* = 3 independent samples (AQP4-IgG +, MOG-IgG +, DSN, and HC). Statistical analysis was performed using one-way ANOVA followed by post hoc testing (*p < 0.05)

WB analyses further supported these observations. Two major AQP4-immunoreactive bands were consistently detected: a lower band (~ 25–37 kDa), corresponding to the M1 and M23 isoforms, and a higher band (~ 50 kDa), corresponding to the extended regulatory isoform of AQP4 (AQP4ex) (Fig. 4F). Quantitative analysis revealed a significant reduction in the M1/M23 band in cells treated with AQP4-IgG+ sera compared with the HC (*p* = 0.0465, Cohen’s *d *= 2.87) (Fig. 4A), while a modest decrease was also observed in the AQP4ex isoform (*p* = 0.1190, Cohen’s *d* = 1.87) (Fig. 4B).

Together, these complementary findings demonstrate that exposure to AQP4-IgG+ sera significantly reduces AQP4 expression in MIO-M1 Müller cells, affecting different isoforms.

##### Kir4.1 Expression

IF analysis revealed a reduction in Kir4.1 signal in MIO-M1 cells treated with AQP4-IgG+ sera compared with those treated with HC sera (*p* = 0.0995), with a very large effect size (Cohen’s *d* = 2.44) (Fig. 3A). Stimulation with MOG-IgG+ or DSN sera did not induce significant changes relative to HC. However, a statistically significant difference was observed between the AQP4-IgG+ and DSN groups (*p* = 0.0493).

WB experiments under the same conditions revealed a similar trend. Kir4.1 protein levels were lower in AQP4-IgG+ sera-treated cells compared with HC (Fig. 4C). Given that Kir4.1 and AQP4 co-localize in Müller cell endfeet as part of the dystrophin-associated protein complex (DAPC), the parallel downregulation of Kir4.1 and AQP4 supports the concept of a functionally linked water-ion regulatory complex in Müller cells, wherein antibody-mediated AQP4 disruption may decrease Kir4.1 expression and localization in AQP4-IgG + conditions [19].

##### CRALBP Expression

IF analysis revealed a slight, non-significant increase in CRALBP signal intensity in cells treated with AQP4-IgG + sera (*p* = 0.1666, Cohen’s *d* = 1.91), whereas stimulation with MOG-IgG+ or DSN sera did not produce detectable changes (Fig. 3A). WB analysis did not reliably detect CRALBP protein under the conditions used. Despite this technical limitation, IF findings suggest a potential gliotic response that warrants further investigation using complementary markers and optimized detection strategies.

##### VEGF Expression

IF analysis demonstrated a significant increase in VEGF signal intensity (*p* = 0.0473) in MIO-M1 cells treated with DSN sera, with a very large effect size (Cohen’s *d* = 2.65) (Fig. 3A). In contrast, VEGF levels in cells exposed to AQP4-IgG+ or MOG-IgG+ sera did not differ significantly from controls. However, a statistically significant difference was observed between MOG-IgG+ and DSN groups (*p* = 0.0039).

WB analysis yielded a similar pattern, although no statistically significant differences in VEGF protein levels among the groups were identified (Fig. 4D). However, DSN-treated cells showed a consistent trend toward increased VEGF expression compared with HC (Cohen’s *d* = 1.49), whereas AQP4-IgG+ and MOG-IgG+ groups remained comparable to baseline.

Although these changes did not reach statistical significance, the concordant trends observed in both IF and WB analyses suggest that DSN patient sera may influence VEGF expression in Müller cells, a finding that needs further investigation with a larger sample size.

##### IL-6 Expression

IF analysis revealed no significant change in IL-6 signal intensity in MIO-M1 cells treated with AQP4-IgG+ (Cohen’s d = 1.23), MOG-IgG+, or DSN sera compared with HC (Fig. 3A). Likewise, WB quantification showed no statistically significant difference in IL-6 protein levels across any of the experimental groups (Fig. 4E). These findings indicate that 12 h serum exposure may not markedly affect intracellular IL-6 accumulation in MIO-M1 cells under BFA-induced retention conditions.

#### AQP4 Localization and Internalization in MIO-M1 Cells Stimulated with Different NMOSD Patient Sera

As shown in Fig. 3B, AQP4–WGA colocalization showed a modest decrease in MIO-M1 cells stimulated with AQP4-IgG+ sera compared with HC (*p* = 0.227, Cohen’s *d* = 1.425), consistent with a mild reduction of membrane-associated AQP4. Conversely, AQP4–EEA1 colocalization exhibited a mild increase (*p* = 0.3497, Cohen’s *d* = 1.058), suggesting enhanced association of AQP4 with early endosomal compartments under the same conditions. Together, these results suggest that exposure to AQP4-IgG+ sera may initiate early endosomal trafficking of AQP4 in MIO-M1 cells.

### Gene Expression Analysis by RT-qPCR

RT-qPCR analysis was performed to assess the transcriptional response of MIO-M1 cells following serum stimulation from different patient groups and HCs.

**Fig. 5:**
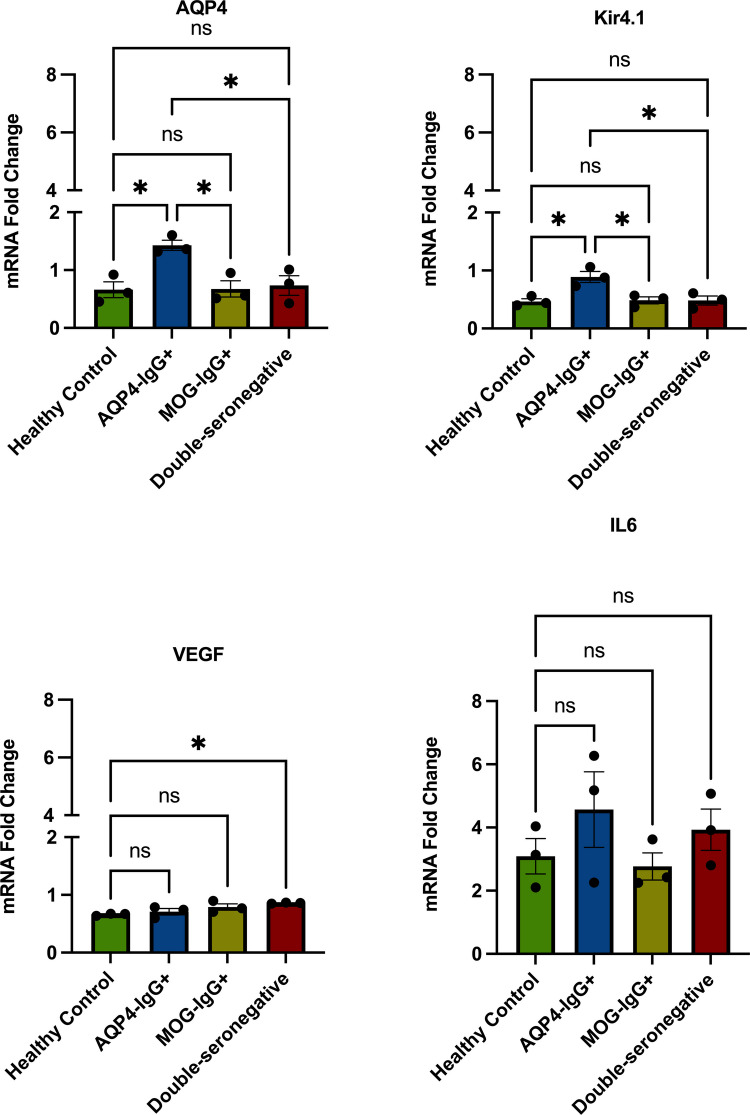
Gene expression analysis of MIO-M1 cells following stimulation with sera from NMOSD patient groups and HCs. MIO-M1 cells were stimulated for 12 h at 37 °C with 20% heat-inactivated sera from AQP4-IgG+, MOG-IgG+, DSN patients, and HC. RT-qPCR was performed to assess the relative expression of AQP4, Kir4.1, VEGF, and IL-6 genes. Expression levels were normalized to GAPDH and presented as relative fold-changes compared to healthy controls. A significant increase in AQP4 and Kir4.1 mRNA expression was detected in cells treated with AQP4-IgG+ sera, while VEGF expression was elevated in DSN-stimulated cells. Data are presented as mean ± SEM. Each group included *n* = 3 independent samples (AQP4-IgG+, MOG-IgG+, DSN, and HC). Statistical analysis was performed using one-way ANOVA followed by post hoc testing (*p < 0.05)

As shown in Fig. 5, stimulation of MIO-M1 cells with AQP4-IgG + sera resulted in a significant upregulation of AQP4 mRNA expression (*p* = 0.0173), with an extremely large effect size (Cohen’s *d* = 3.24). This finding contrasts with the IF and WB results, which demonstrated a reduction in AQP4 protein levels. Our experiments pointed out compensatory transcriptional upregulation in response to AQP4-IgG–mediated protein loss, reflecting the cells’ attempt to restore AQP4 homeostasis. Moreover, significant differences were observed between the AQP4-IgG+ and MOG-IgG+ groups (*p* = 0.0191, Cohen’s *d* = 3.18) and between the AQP4-IgG+ and DSN groups (*p* = 0.0290, Cohen’s *d* = 2.93).

Kir4.1 mRNA expression was likewise upregulated in MIO-M1 cells following stimulation with AQP4-IgG+ sera (*p* = 0.0141), with a large effect size (Cohen’s *d* = 3.36) (Fig. 5), whereas treatment with MOG-IgG+ or DSN sera did not induce significant changes. This coordinated upregulation of AQP4 and Kir4.1 transcription supports the presence of a shared compensatory response under antibody-mediated effects. Moreover, significant differences were observed between the AQP4-IgG + and MOG-IgG + groups (*p* = 0.0182, Cohen’s *d* = 3.20) and between the AQP4-IgG+ and DSN groups (*p* = 0.0174, Cohen’s *d* = 3.23), further highlighting the distinct transcriptional signature induced by AQP4-IgG+ sera.

CRALBP transcripts could not be detected in any of the experimental groups by RT-qPCR, likely due to technical limitations encountered during the amplification process.

VEGF mRNA expression was notably elevated in cells stimulated with DSN sera (*p* = 0.0366), with a large effect size (Cohen’s *d* = 2.79) (Fig. 5), whereas no significant changes were detected following stimulation with AQP4-IgG+ or MOG-IgG+ sera. This pattern, observed both at the protein and transcript levels, suggests that DSN sera may influence angiogenic signaling through factors independent of AQP4 or MOG antibodies. IL-6 mRNA expression exhibited a modest upward trend in AQP4-IgG+ sera-stimulated cells (Cohen’s *d *= 1.11), and overall, RT-qPCR results did not reveal prominent alterations in IL-6 levels across the experimental groups.

## Discussion

Our findings indicate that exposure of human MIO-M1 Müller cells to AQP4-IgG+ sera leads to a significant reduction in total AQP4 protein expression, as evidenced by both IF and WB analyses. AQP4 exists mainly in two isoforms, M1 and M23, which differ at their N-termini and determine the formation of higher-order orthogonal arrays of particles (OAPs) in the plasma membrane. While M23 efficiently forms OAPs, M1 limits their size due to palmitoylation at cysteine residues 13 and 17. These OAPs cluster AQP4 molecules into organized lattices, increasing the density of extracellular epitopes recognized by AQP4-IgG, thereby enhancing antibody binding and complement activation in NMOSD [20]. In our experiments, two major AQP4-immunoreactive bands were consistently detected: a lower band (~ 25–37 kDa) corresponding to the classical M1-M23 isoforms, and a higher band (~ 50 kDa) corresponding to the translational read-through isoform AQP4ex [21]. The lower band showed a significant reduction following AQP4-IgG+ sera treatment.

Interestingly, the AQP4ex isoform implicated in stabilizing AQP4 at the plasma membrane [21] exhibited a modest reduction following AQP4-IgG+ sera treatment. AQP4ex interacts with α-syntrophin and anchors AQP4 to the DAPC preserving its polarized localization and OAPs at astrocyte endfeet [22, 23]. Loss of AQP4ex may disrupt this organization, leading to AQP4 mislocalization, altered α-syntrophin expression, and reduced perivascular confinement. Together, these observations might suggest that decreased AQP4ex in AQP4-IgG+ treated MIO-M1 cells may compromise the membrane stability of AQP4 and facilitate its redistribution from the plasma membrane to intracellular compartments.

To explore AQP4 redistribution, we performed colocalization analyses to measure membrane-bound and intracellular AQP4 localization. AQP4–WGA colocalization slightly decreased in MIO-M1 cells stimulated with AQP4-IgG+ sera compared with the HC, suggesting a possible reduction of membrane-associated AQP4. Conversely, AQP4–EEA1 colocalization displayed a mild increase, which may indicate a possible redistribution of AQP4 toward early endosomal compartments. Although these changes did not reach statistical significance, the observed trends are consistent with the previous findings of Netti et al. [24], who demonstrated that AQP4-IgG exposure leads to a decrease in AQP4–WGA membrane colocalization and a concomitant increase in AQP4–EEA1 overlap, suggesting antibody-induced internalization of AQP4 into endosomal vesicles. Although direct surface quantification assays such as flow cytometry were not performed, our combined immunofluorescence, Western blot, and colocalization analyses provide evidence consistent with altered AQP4 localization and potential intracellular redistribution following exposure to AQP4-IgG+ sera. Nevertheless, surface-specific assays such as flow cytometry or biotinylation approaches would further strengthen the direct assessment of membrane AQP4 dynamics and should be considered in future studies. These observations may therefore reflect an early, complement-independent phase of AQP4 trafficking initiated by antibody engagement.

In the present study, patient sera were heat-inactivated to eliminate complement activity. While complement-dependent cytotoxicity represents a central mechanism of astrocyte injury in NMOSD mediated by AQP4-IgG–induced complement activation [25], the use of heat-inactivated serum allowed us to specifically investigate complement-independent antibody-mediated effects on retinal Müller cells. This approach enabled the evaluation of direct cellular responses to patient antibodies without the confounding effects of complement-mediated cytotoxicity, which could otherwise obscure more subtle processes such as AQP4 redistribution, intracellular trafficking, or cellular stress responses. Importantly, accumulating evidence indicates that AQP4-IgG can exert pathogenic effects independent of complement activation, including AQP4 clustering, endocytosis, and cytokine release from astrocytes in vitro. Consistent with this concept, experimental studies have demonstrated that intravitreal delivery of AQP4-IgG in animal models results in antibody deposition on retinal Müller cells, reduced retinal AQP4 expression, increased GFAP expression, and progressive retinal ganglion cell loss even in the absence of detectable complement activation [26]. Therefore, our complement-free experimental system allowed us to specifically examine antibody-driven alterations in Müller cell biology, including changes in AQP4 expression, Kir4.1 regulation, and VEGF signaling, which may represent early or complement-independent mechanisms contributing to retinal pathology in NMOSD. Nevertheless, future studies incorporating active complement systems and more complex co-culture models will be important to better recapitulate the in vivo inflammatory microenvironment.

This study demonstrated that, in addition to AQP4-IgG+ sera, both MOG-IgG+ and DSN sera induce a significant reduction in Müller cell metabolic activity. Given that MOG is not a canonical Müller cell surface target in the same way that AQP4 is relevant to glial biology, this effect is unlikely to be mediated by direct antibody–cell interactions. Instead, the observed reduction in MTT signal in the MOG-IgG+ group likely reflects indirect effects mediated by circulating inflammatory components present in patient sera, including cytokines, immune complexes, extracellular vesicles, or other soluble mediators causing neuroinflammation [27]. The sustained suppression of metabolic activity from 24 h onward further suggests that MOG-IgG+ sera can compromise Müller cell function through a stable, non-progressive mechanism. In contrast, DSN sera induced a significant but comparatively less pronounced reduction in metabolic activity. In the absence of detectable AQP4 or MOG antibodies, these effects are likely mediated by alternative pathogenic mechanisms similar to MOG or unidentified autoantibodies. The intermediate magnitude of the response in the DSN group may reflect the biological heterogeneity of this patient population.

Antibody engagement with structured extracellular AQP4 epitopes is thought to trigger internalization and altered trafficking of AQP4 [28, 29], which could explain the observed reduction in total protein levels despite a compensatory upregulation of AQP4 mRNA expression in our RT-qPCR results. This transcriptional upregulation likely reflects a possible feedback response to antibody-mediated protein loss, which should be validated with further mechanistic studies. Similar observations have been reported in neuropathological investigations in NMO lesions, where astrocytes lacking surface AQP4 protein display preserved or even elevated AQP4 mRNA transcripts, indicating that AQP4 depletion is not driven by gene downregulation but rather by IgG-induced internalization and degradation of the protein. In these lesions, reactive astrocytes exhibit strong AQP4 mRNA signals despite minimal membrane-bound AQP4, supporting the notion that surviving astrocytes attempt to restore physiological function through enhanced transcriptional synthesis of AQP4 [30]. Similarly, in our MIO-M1 cells, the increase in AQP4 transcript abundance likely reflects an intrinsic feedback mechanism aimed at reestablishing membrane AQP4 homeostasis following antibody-mediated endocytosis.

The concurrent decrease in AQP4 and Kir4.1 protein expression observed across our IF and WB, together with the compensatory mRNA upregulation detected in our RT-qPCR experiments following AQP4-IgG+ sera stimulation, underscores the structural and functional interdependence of these channels within Müller cells. Functionally, this coordinated downregulation may suggest a tightly coupled regulatory mechanism between the two channels that is essential for maintaining ionic and osmotic homeostasis in retinal glia. Although the direct functional coupling between AQP4 and Kir4.1 has been debated, physiological evidence supports their interdependence. For example, Aqp4 knockout mice exhibit reduced potassium uptake and altered neuronal excitability [31], underscoring the cooperative regulation of osmolarity by these channels. Additional evidence for their functional coupling arises from disease models. For instance, Lassiale et. al. reported that in inherited retinal dystrophy, both AQP4 and Kir4.1 are mislocalized and downregulated in Müller cell endfeet, leading to impaired potassium siphoning and retinal edema [32]. Structurally, this interdependence is mediated by their common connection to the DAPC particularly through the DP71 isoform. AQP4 and Kir4.1 co-cluster at Müller cell endfeet by binding to the DAPC, and light-microscopic studies have confirmed their colocalization. Importantly, loss of AQP4 disrupts DP71, which in turn destabilizes the entire DGC, leading to mislocalization of both channels [19]. Furthermore, such a disturbance in AQP4–Kir4.1 coupling could impair extracellular potassium clearance during neuronal activity, contributing to hyperexcitability, cytotoxic edema, and glial dysfunction, pathological features observed not only in NMOSD but also in a variety of neuropsychiatric conditions [33]. Therefore, AQP4-IgG–mediated loss of AQP4 in Müller cells may not only impair osmoregulatory capacity but also reduce Kir4.1-mediated potassium buffering, thereby amplifying retinal excitotoxicity and inflammation.

In our experiments at both transcript and protein levels, VEGF expression exhibited a trend toward upregulation, particularly in MIO-M1 cells stimulated with DSN sera, the subgroup whose pathophysiology remains least characterized. This finding suggests that alternative, non-antibody-dependent mechanisms may contribute to Müller cell activation and angiogenic signaling in antibody-negative NMOSD. Elevated VEGF expression in glial cells has been widely associated with retinal and CNS responses to stress and injury. In ophthalmic disorders such as age-related macular degeneration, proliferative diabetic retinopathy, and neovascular glaucoma, chronic VEGF upregulation promotes pathological angiogenesis and breakdown of the blood–retina barrier [34]. However, several studies have also reported that transient VEGF expression in Müller cells can occur as part of a glial or neuronal stress response, reflecting an attempt to maintain vascular integrity or support cell survival [35, 36]. Similarly, astrocytes are known to release VEGF in the context of neuroinflammation and ischemic injury, where it may contribute to both repair and secondary inflammatory processes [37]. Therefore, the VEGF elevation observed in DSN-treated Müller cells may represent a complex response that integrates inflammatory and potentially protective pathways. Further studies assessing VEGF secretion kinetics and associated cytokine profiles are required to clarify whether this upregulation primarily reflects a pathogenic or compensatory mechanism in antibody-negative NMOSD.

A growing body of OCT studies has revealed the retinal involvement in NMOSD, which is not only limited to secondary retrograde degeneration following optic nerve damage but also includes primary retinopathy. Thinning of the pRNFL and GCIPL has been consistently observed in AQP4-IgG+ NMOSD eyes, not only after ON episodes but also in eyes without any ON history, suggesting the presence of primary retinopathy independent of optic nerve inflammation [8, 38–40]. This primary pathology is hypothesized to involve AQP4-IgG-mediated damage to astrocytes and Müller cells. In a retinal explant model, Wolf et al. demonstrated that NMOSD-derived IgG can directly interact with retinal tissue and induce Müller cell activation, characterized by increased GFAP expression and alterations in local chemokine secretion. These findings indicate that retinal glial cells may directly respond to pathogenic antibodies in NMOSD and develop stress responses independent of optic nerve inflammation. Collectively, these observations suggest that NMOSD IgG–induced Müller cell dysfunction may contribute to retinal degeneration and may represent one potential mechanism underlying the retinal thinning reported in NMOSD patients [41]. Such findings align with our results, where AQP4-IgG + sera was associated with AQP4 downregulation with possible internalization in Müller cells in vitro, independent of complement activation.

Zeka et al. demonstrated that intraretinal injection of AQP4-specific T cells in rodents resulted in marked T-cell infiltration into the inner retinal layers, causing Müller cell, axonal, and neuronal injury even in the absence of AQP4-IgG [42]. These findings suggest that antibody-independent, T cell–mediated mechanisms may also contribute to NMOSD-associated retinopathy. This possibility is particularly relevant to the DSN subgroup, in which no pathogenic circulating antibodies have been identified, yet retinal changes are frequently observed [43]. In our study, the increase in VEGF expression observed in the DSN group may reflect ischemic stress secondary to T cell–mediated Müller cell damage, which could activate pro-angiogenic pathways as a compensatory response to tissue injury.

This study has several limitations that should be taken into account when interpreting the findings. Firstly, the relatively small sample size due to the rarity of the disease limits the statistical power and generalizability of our results. Our findings provide exploratory evidence and should be interpreted as hypothesis-generating. Future studies with larger patient cohorts are necessary to validate the observed trends and strengthen the conclusions. Another limitation of our current study is that we were unable to distinguish between the M1 and M23 isoforms of AQP4, which may have differential roles in membrane localization and susceptibility to antibody-mediated internalization. In future experiments, we plan to use isoform-specific AQP4 antibodies to analyze the expression and distribution of M1 and M23 isoforms separately, thereby providing a more precise understanding of which isoform is preferentially affected by AQP4-IgG.

## Conclusion

This study shows that sera from AQP4-IgG+ NMOSD patients can directly act on human Müller glial cells, inducing a complement-independent loss of AQP4, which affects both the major M1/M23 pool and, to a lesser extent, the membrane-stabilizing AQP4ex isoform. This loss was accompanied by parallel reductions in Kir4.1 protein and compensatory upregulation of both AQP4 and Kir4.1 mRNA, suggesting that antibody binding triggers internalization/degradation first, followed by transcriptional responses later. Together, these changes point to an early disturbance of the AQP4–Kir4.1/DAPC water–ion buffering unit, a mechanism that could underlie the primary retinal involvement described in AQP4-IgG+ NMOSD. In contrast, sera from DSN patients preferentially increased VEGF expression, implying that Müller cell activation can also be driven by antibody-independent serum factors associated with inflammation or ischemia. These findings position Müller cells as active contributors to NMOSD-associated retinal pathology rather than passive bystanders. By using the Müller cells for the first time across AQP4-IgG+, MOG-IgG+, and DSN-NMOSD subgroups, we provide experimental support for subgroup-specific retinal glial responses and offer a tractable platform to dissect patient-derived mechanisms of NMOSD retinopathy. Larger cohorts and isoform-specific AQP4 tools are needed to confirm these pathways to clarify whether Müller cell–directed interventions could help preserve retinal integrity in NMOSD.

## Supplementary Information

Below is the link to the electronic supplementary material.

ESM 1 DOCX (23.6 MB)

## Data Availability

The data supporting the findings of this study are included in the article. Raw experimental data generated during the current study are available from the corresponding author upon reasonable request.

## Abbreviations

NMOSD: Neuromyelitis optica spectrum disorder
CNS: Central nervous system
ON: Optic neuritis
LETM: Longitudinally extensive transverse myelitis
APS: Area postrema syndrome
AQP4: Aquaporin-4
AQP4-IgG: Aquaporin-4 immunoglobulin G
AQP4ex: Aquaporin-4 extended isoform
MOG: Myelin oligodendrocyte glycoprotein
MOG-IgG: Myelin oligodendrocyte glycoprotein immunoglobulin G
MOGAD: Myelin oligodendrocyte glycoprotein antibody-associated disease
DSN: Double-seronegative
Kir4.1: Inwardly rectifying potassium channel 4.1
DAPC: Dystrophin-associated protein complex
DP71: Dystrophin protein 71
OAPs: Orthogonal arrays of particles
GCIPL: Ganglion cell–inner plexiform layer
pRNFL: Peripapillary retinal nerve fiber layer
BRB: Blood–retina barrier
BBB: Blood–brain barrier
CSF: Cerebrospinal fluid
VEGF: Vascular endothelial growth factor
IL-6: Interleukin-6
CRALBP: Cellular retinaldehyde-binding protein
MIO-M1: Moorfields/Institute of Ophthalmology-Müller 1 cell line
HC: Healthy control
IPND: International Panel for NMO Diagnosis
EDSS: Expanded Disability Status Scale
IF: Immunofluorescence
WB: Western blotting
RT-qPCR: Real-time quantitative polymerase chain reaction
WGA: Wheat germ agglutinin
EEA1: Early endosome antigen-1
MOC: Manders’ overlap coefficient
MTT: 3-(4,5-Dimethylthiazol-2-yl)-2,5-diphenyltetrazolium bromide
BFA: Brefeldin A
PBS: Phosphate-buffered saline
PFA: Paraformaldehyde
SFM: Serum-free medium
FBS: Fetal bovine serum
RIPA: Radioimmunoprecipitation assay
BCA: Bicinchoninic acid
PVDF: Polyvinylidene fluoride
ECL: Enhanced chemiluminescence
Ct: Cycle threshold
SD: Standard deviation
SEM: Standard error of the mean
ANOVA: Analysis of variance
