# Supplementary material for: Molecular Mechanisms of Retinal Damage in NMOSD via Müller Glial Cell Stimulation with Patient Sera
Source: Mol Neurobiol. 2026 May 4;63(1):607. doi: 10.1007/s12035-026-05892-y (PMC13139280; doi:10.1007/s12035-026-05892-y)
Supplement: Supplementary file 1 — DOCX (23.6 MB) [file 12035_2026_5892_MOESM1_ESM.docx]

**Prolonged incubation of MIO-M1 cells with AQP4-IgG+ sera resulted in AQP4 downregulation**

Based on previous findings, long-term exposure of MIO-M1 cells to AQP4-IgG+ sera results in downregulation of the AQP4.[19] In this context,we aimed to validate these findings and determine the optimal temperature conditions for our subsequent experiments.To this end,MIO-M1 cells were treated with heat-inactivated AQP4-IgG+ and HC sera(n = 1) under two temperature conditions,following the protocol described by Netti et al.[19]Briefly,cells were incubated for 1h at 4°C to allow antibody binding while preventing active endocytosis,or for 12hrs at 37°C to enable antibody-mediated downregulation.At 4°C,AQP4 IF intensity remained similar between cells treated with sera,indicating that low temperature effectively inhibited AQP4 internalization.In contrast,after 12hrs at 37°C,cells exposed to AQP4-IgG+ sera showed a reduction in AQP4 expression compared with HC-treated cells(Figure 3).This temperature-dependent decrease in AQP4 signal suggests that prolonged incubation at 37°C facilitates antibody-mediated downregulation of AQP4,consistent with the findings of Netti et al.[19]


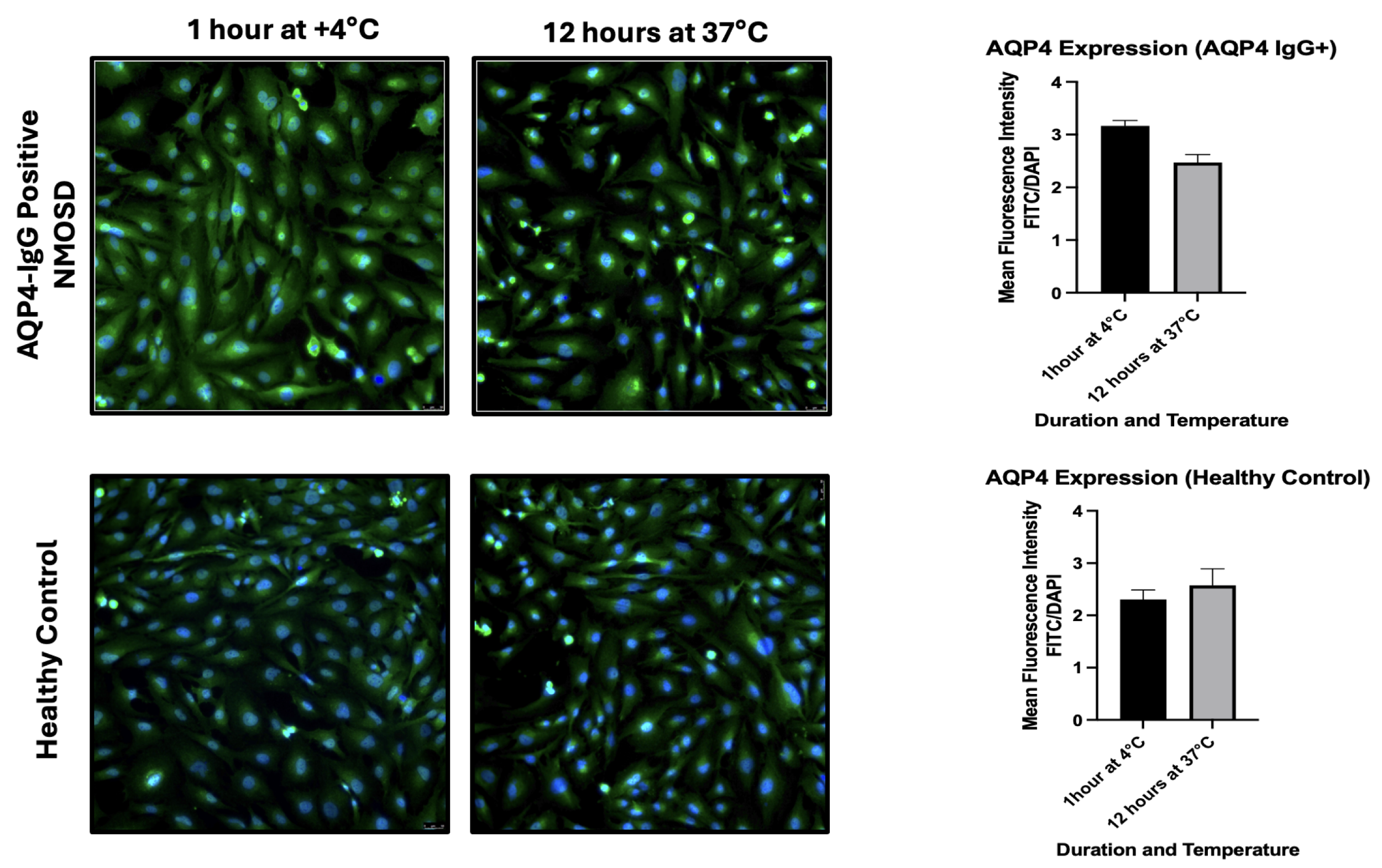


***_Figure X- Downregulation of AQP4 in MIO-M1 cells exposed to AQP4-IgG+ sera._***

_IF images show AQP4 (green) and DAPI (blue) staining after 1 h at 4 °C or 12 h at 37 °C, demonstrating reduction of AQP4 expression at physiological temperature compared to HC sera._

**WESTERN BLOT WHOLE BLOTS BY SETS**

**AQP4 SET 1 with Vinculin**

**
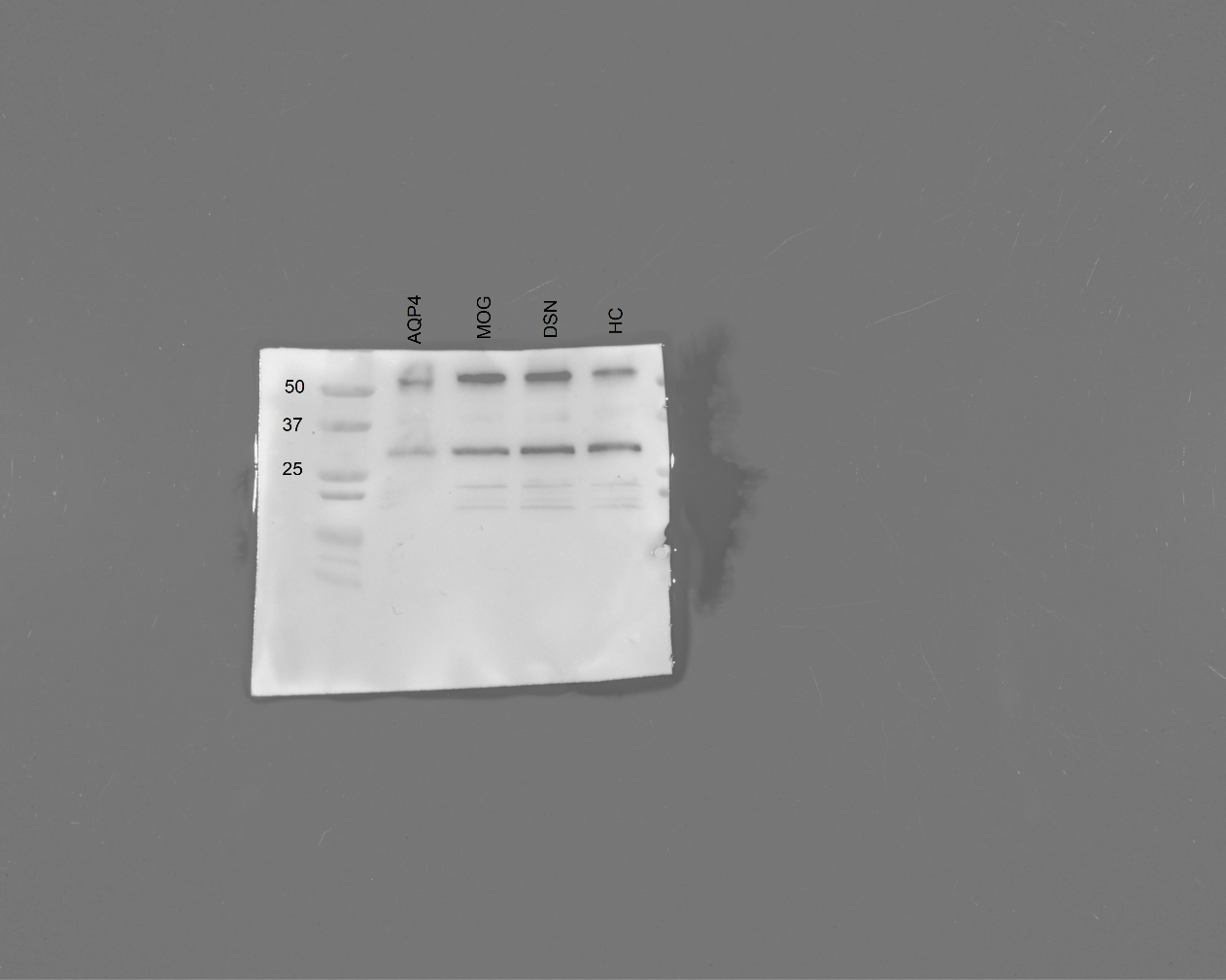
**

**AQP4 with ladder**

**
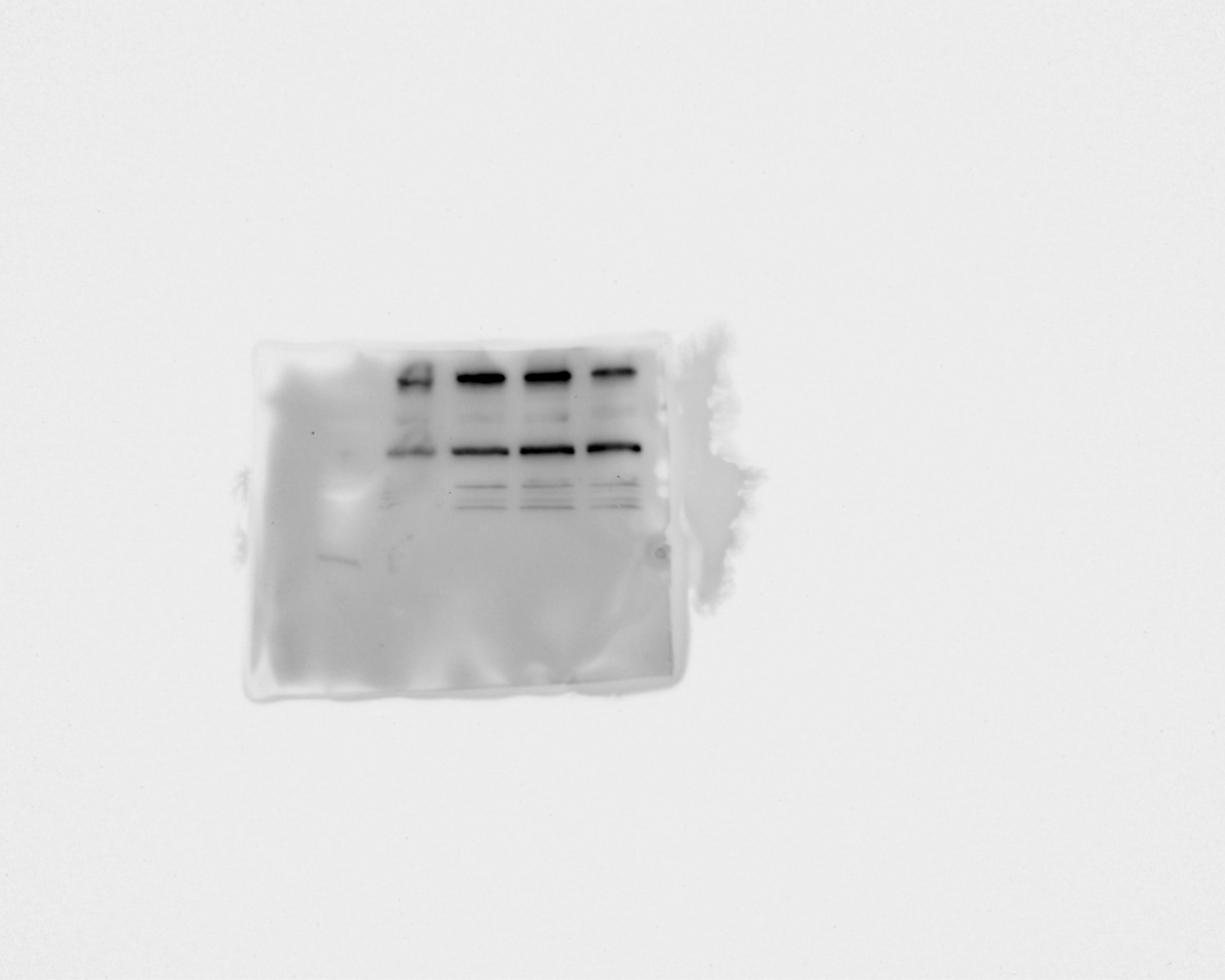
**

**AQP4 without ladder**


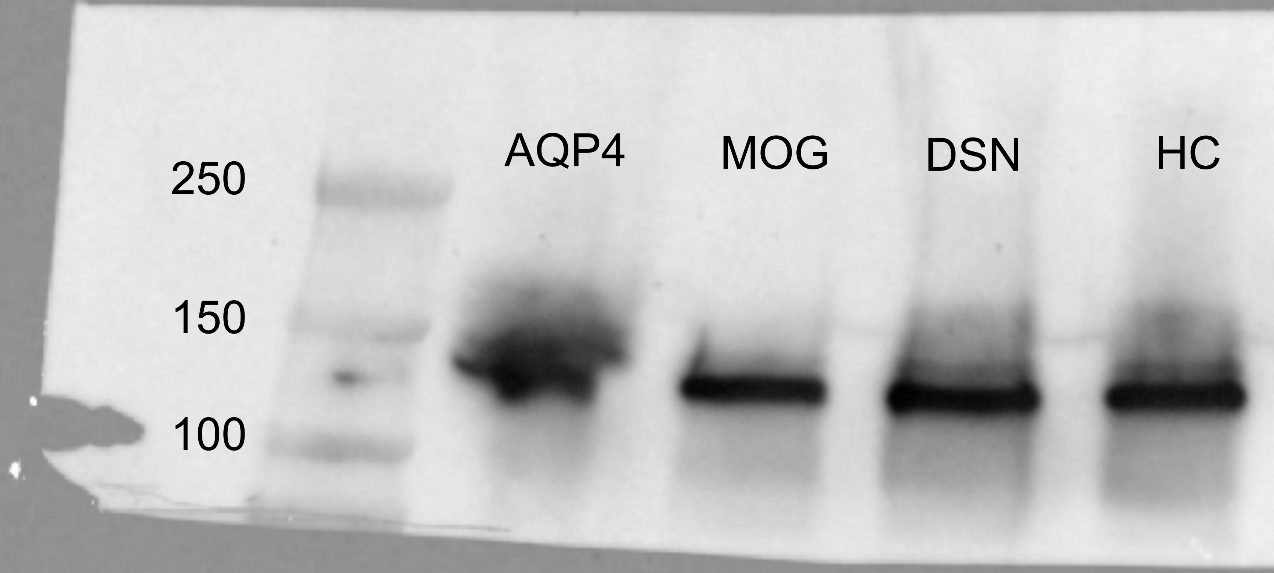


**Vinculin with ladder**

**AQP4 SET 2 with Vinculin**

**
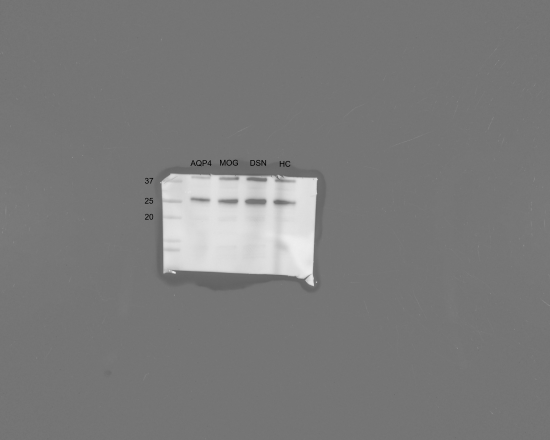
**

**AQP4 with ladder**

**
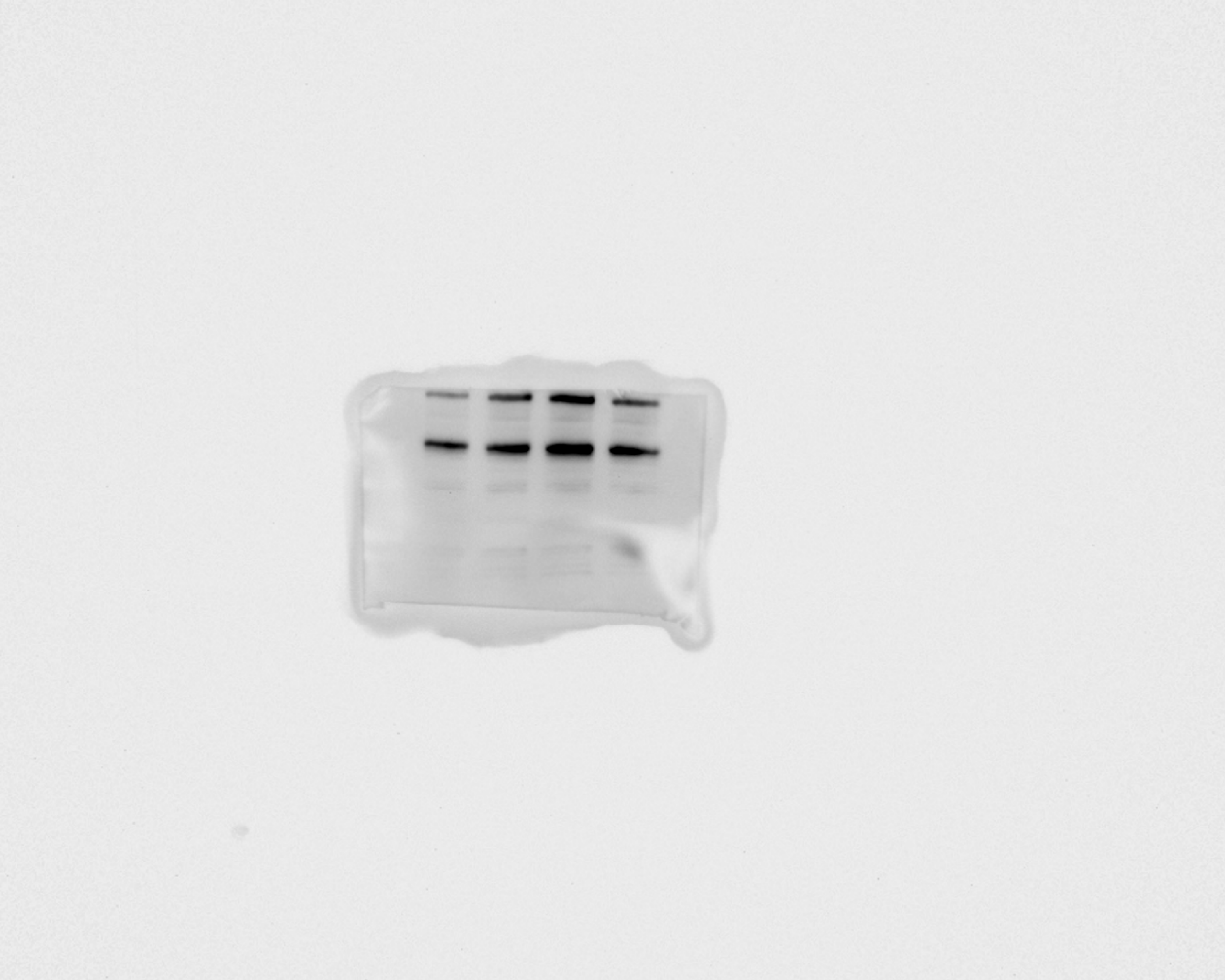
**

**AQP4 without ladder**

**
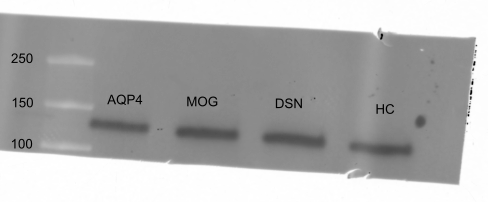
**

**Vinculin with ladder**

**AQP4 SET 3 with Vinculin**

**
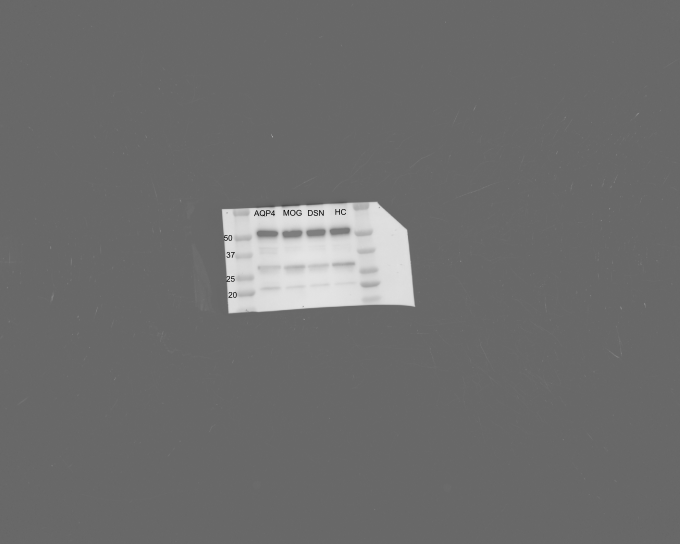
**

**AQP4 with ladder**

**
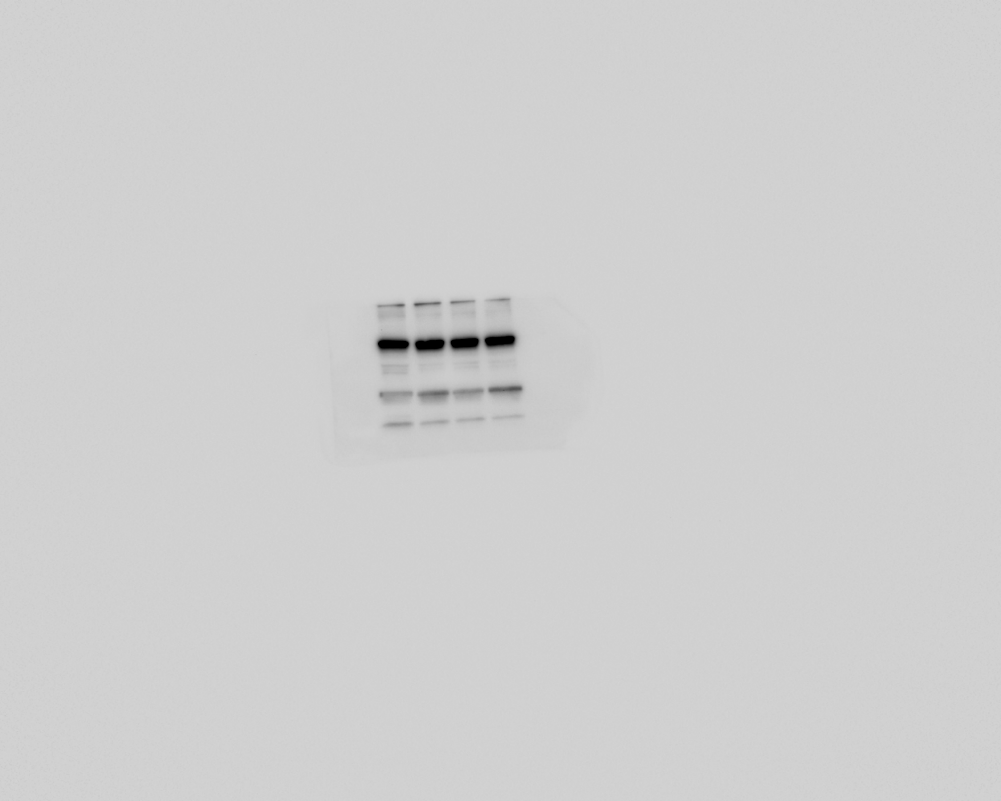
**

**AQP4 without ladder**

**
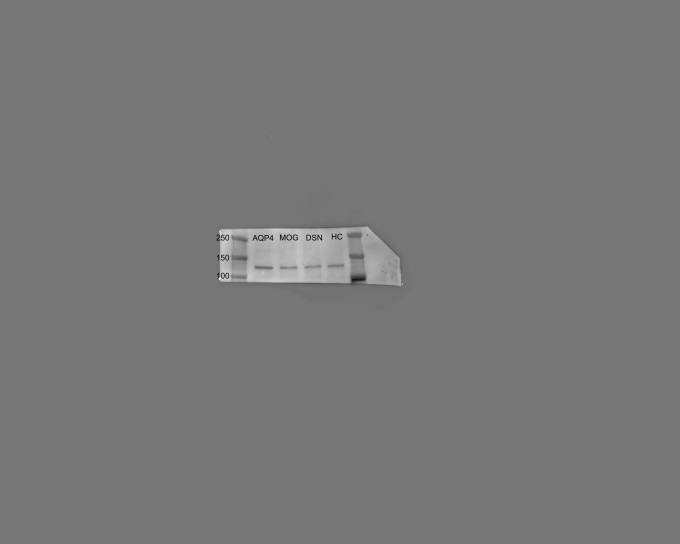
**

**Vinculin with ladder**

**Kir4.1 SET 1 with Vinculin**

**
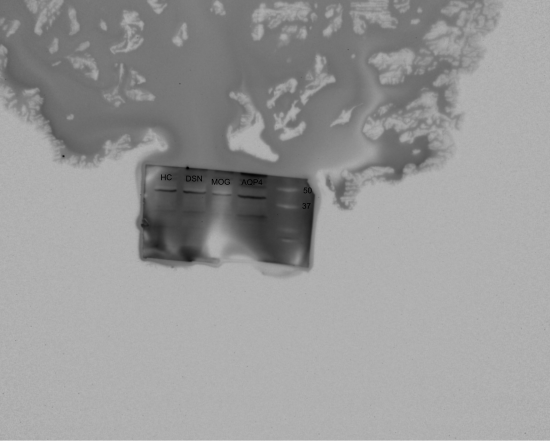
**

**Kir4.1 with ladder**

**
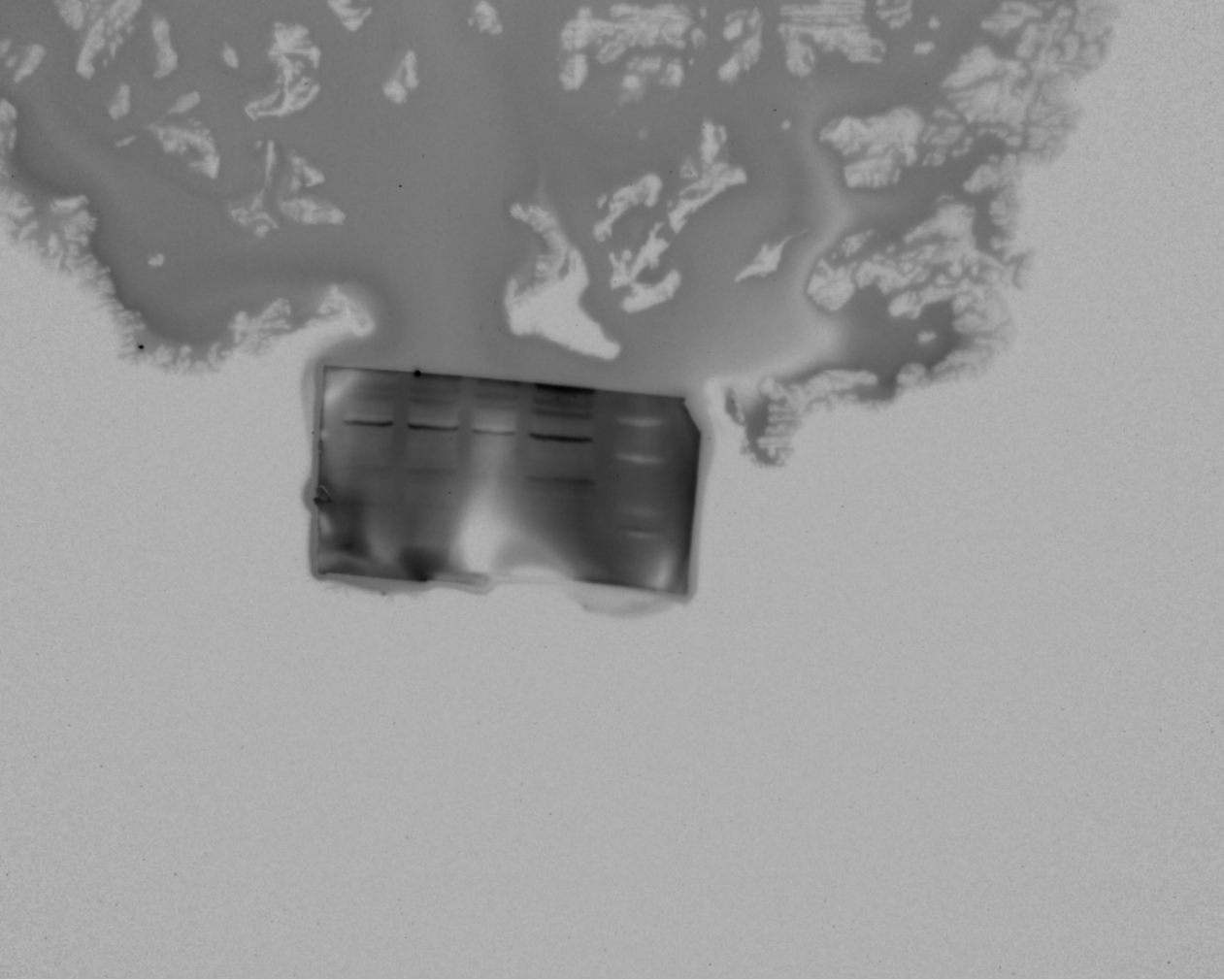
**

**Kir4.1 without ladder**

**
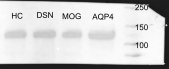
**

**Vinculin with ladder**

**Kir4.1 SET 2 with Vinculin**

**
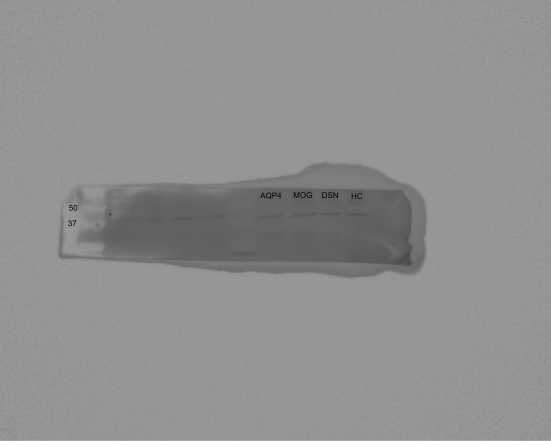
**

**Kir4.1 with ladder**

**
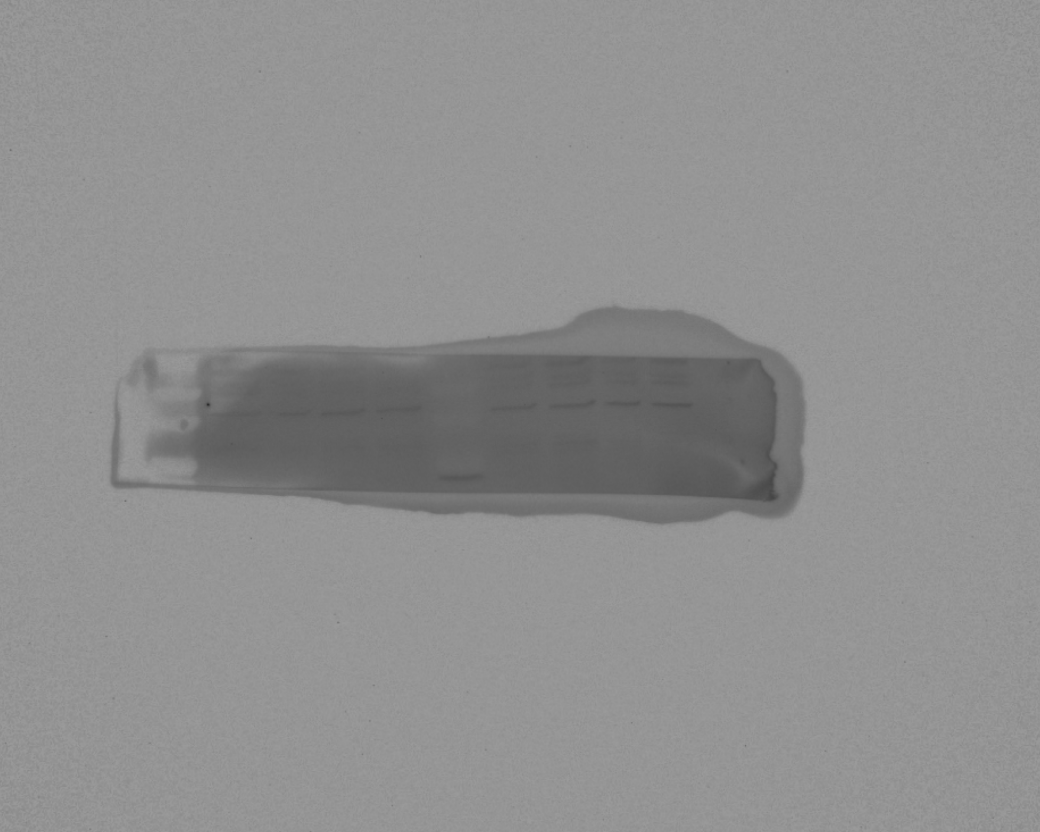
**

**Kir4.1 without ladder**

**
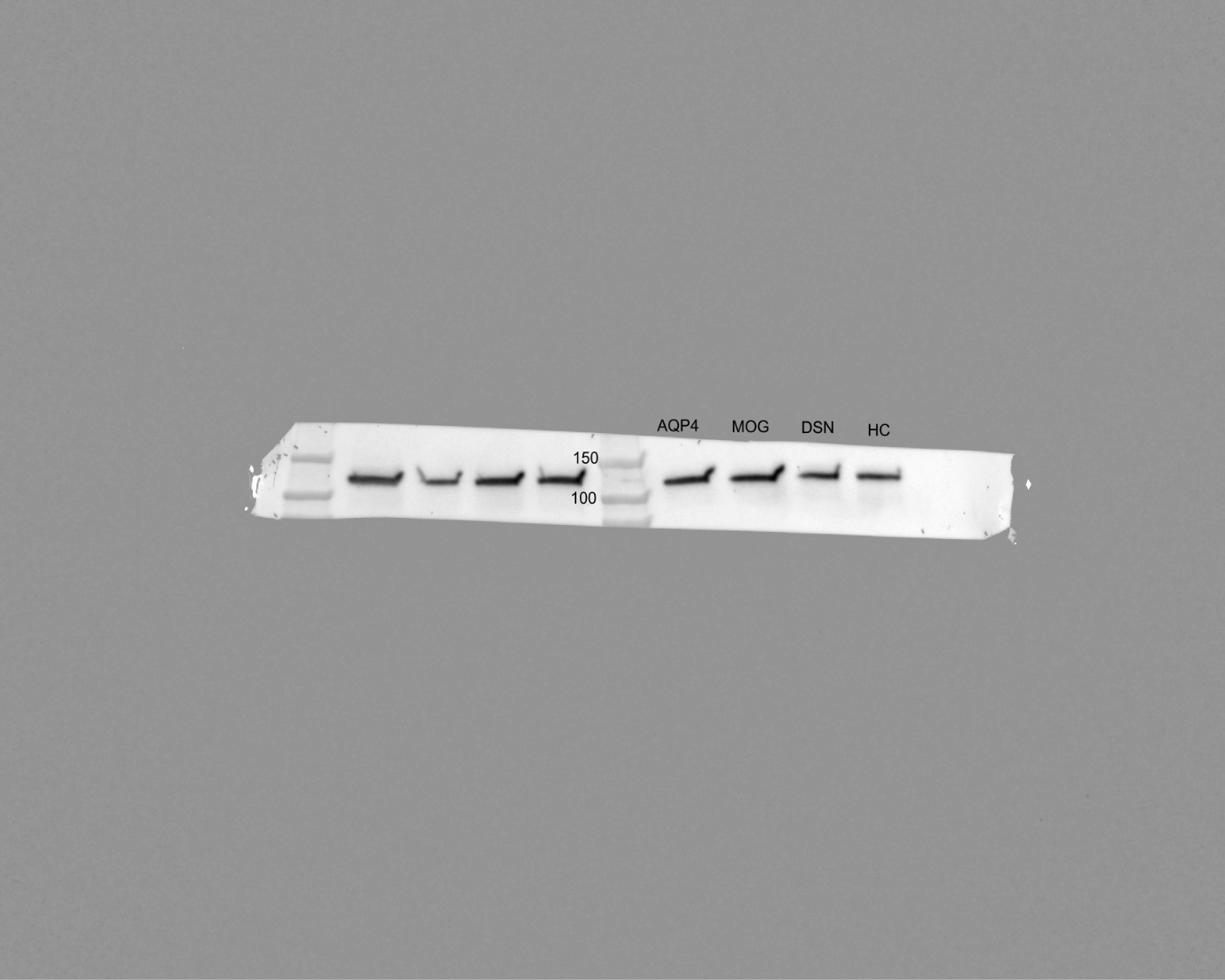
**

**Vinculin with ladder**

**Kir4.1 SET 3 with Vinculin**

**
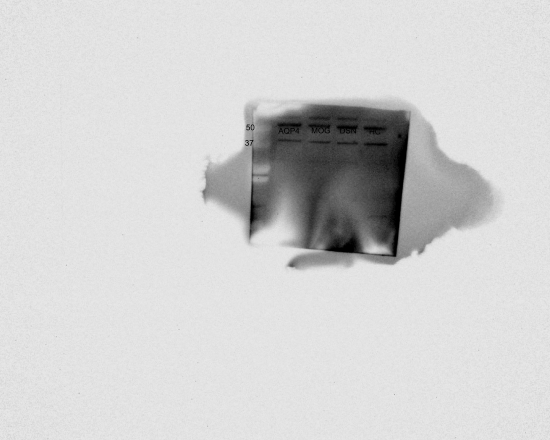
**

**Kir4.1 with ladder**

**
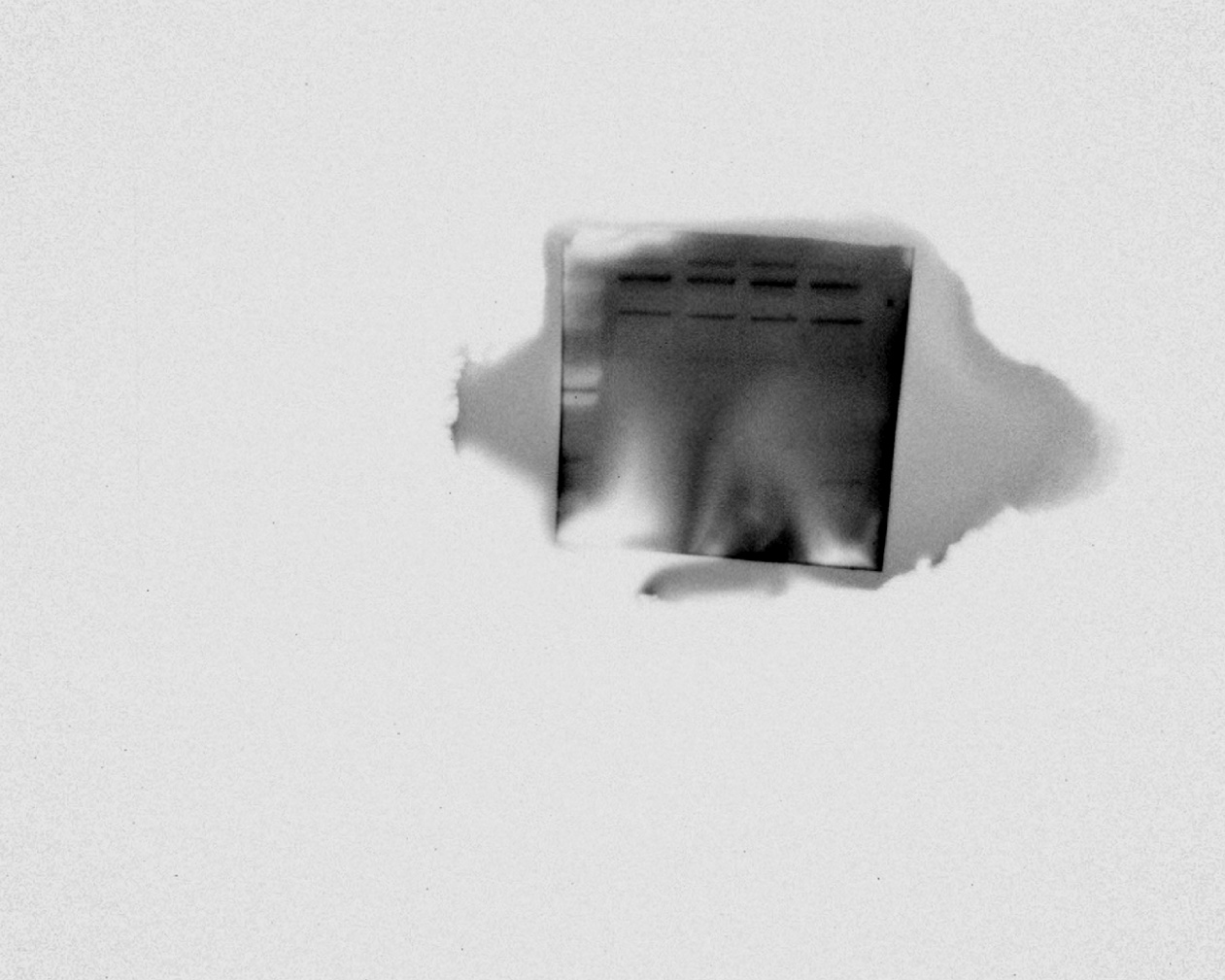
**

**Kir4.1 without ladder**

**
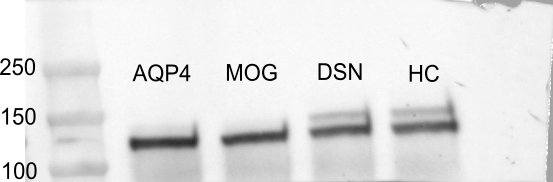
**

**Vinculin with ladder**

**VEGF Set 1 with Vinculin**

**
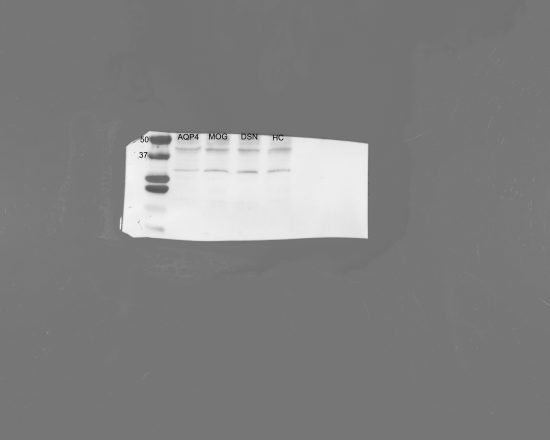
**

**VEGF with ladder**

**
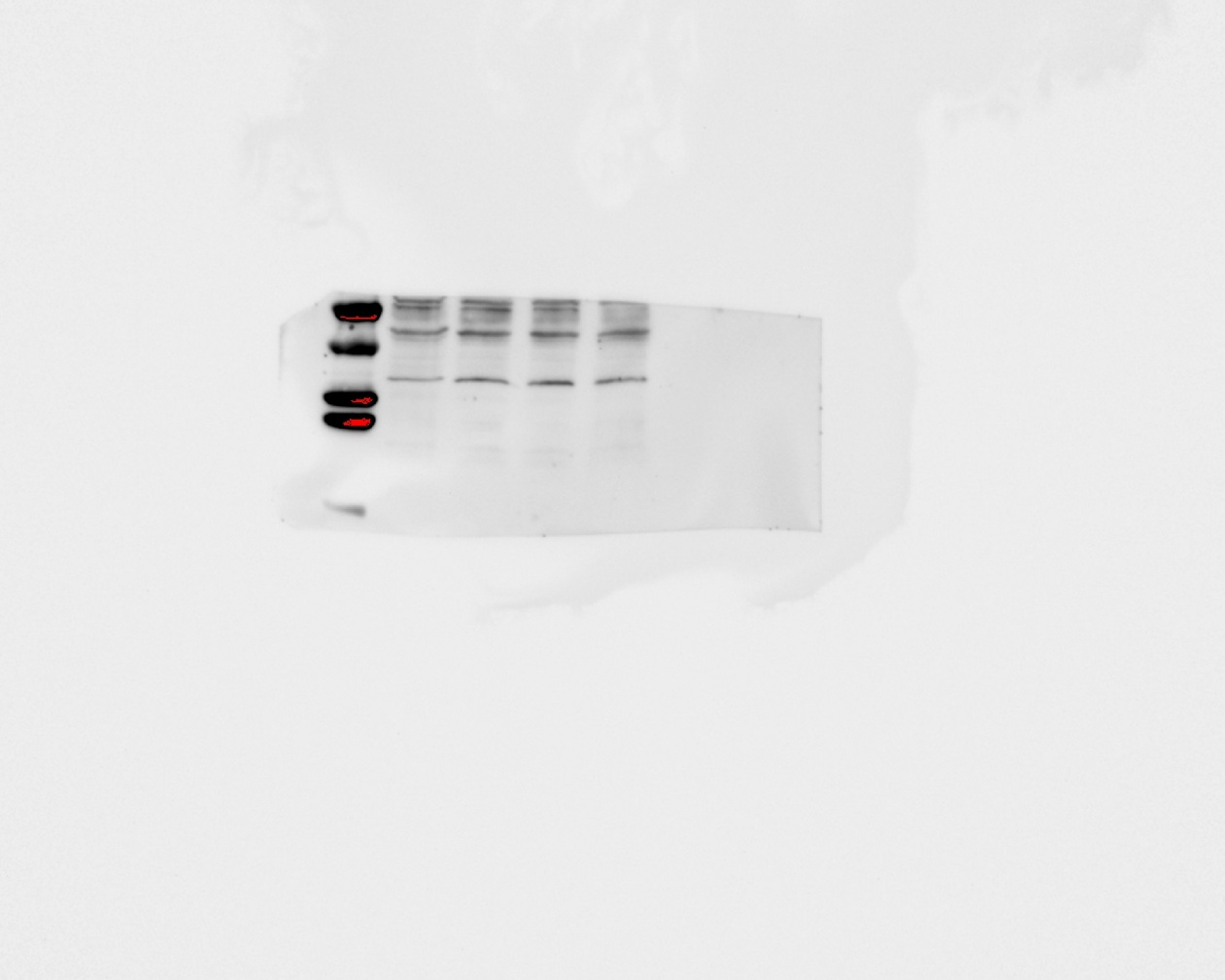
**

**VEGF without ladder**

**
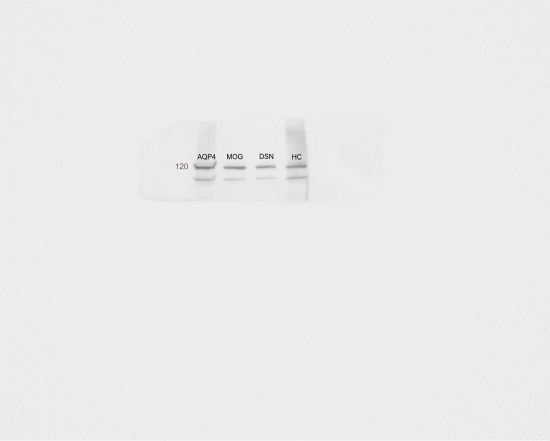
**

**Vinculin**

**VEGF Set 2 with Vinculin**

**
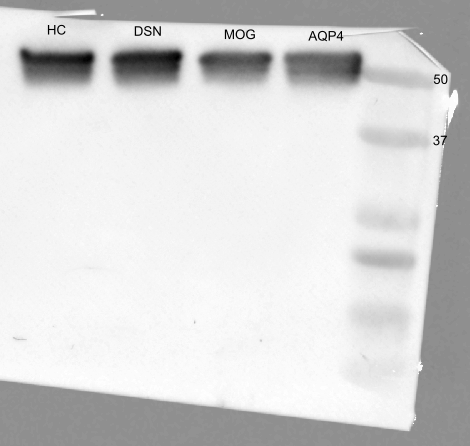
**

**VEGF with ladder**

**
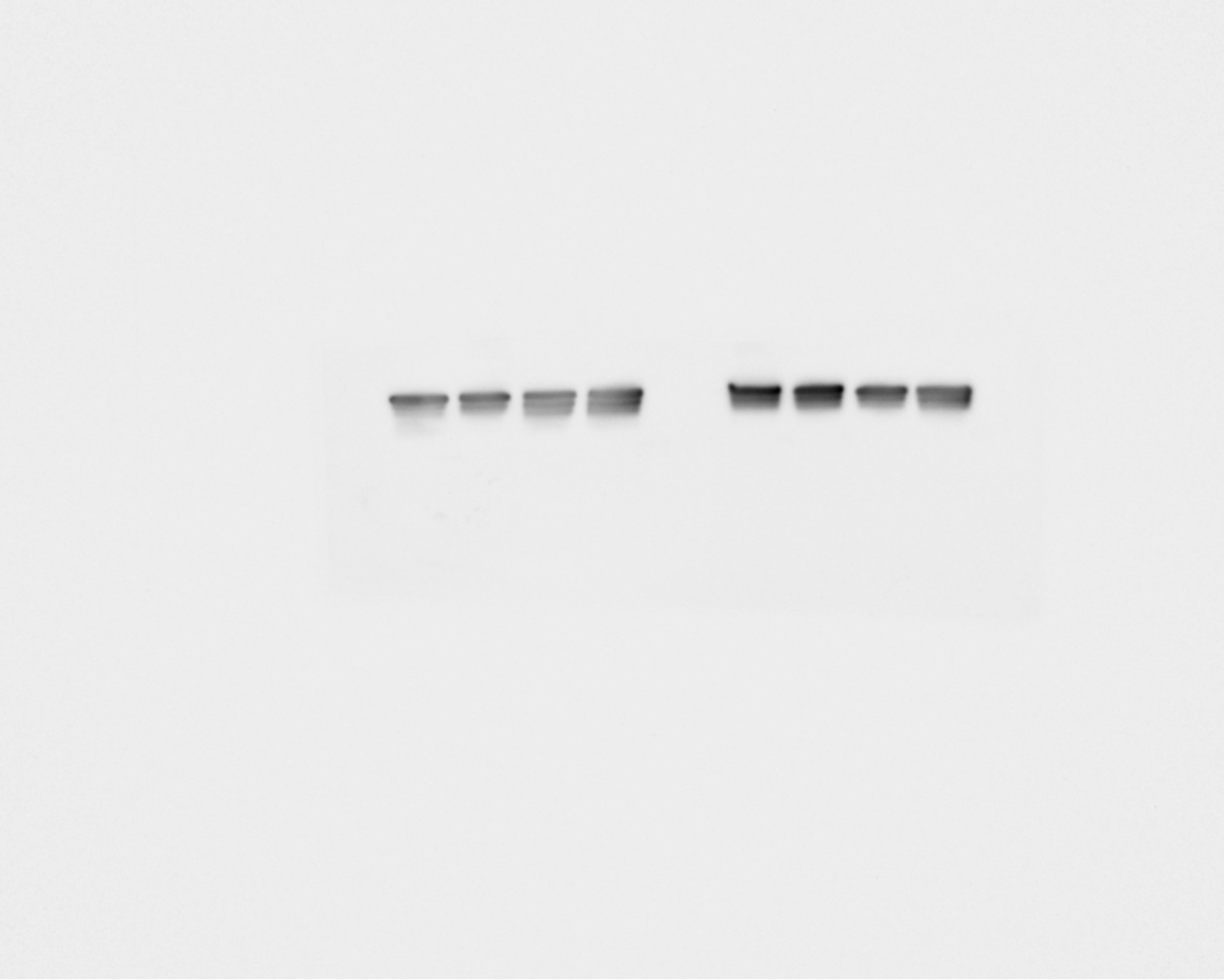
**

**VEGF without ladder**

**
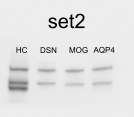
**

**Vinculin**

**VEGF Set 3 with Vinculin**

**
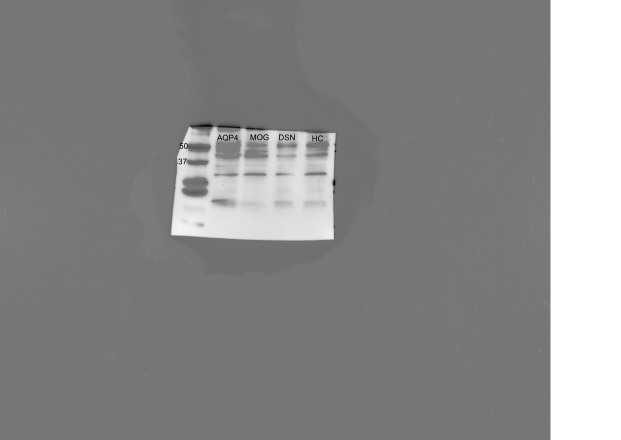
**

**VEGF with ladder**

**
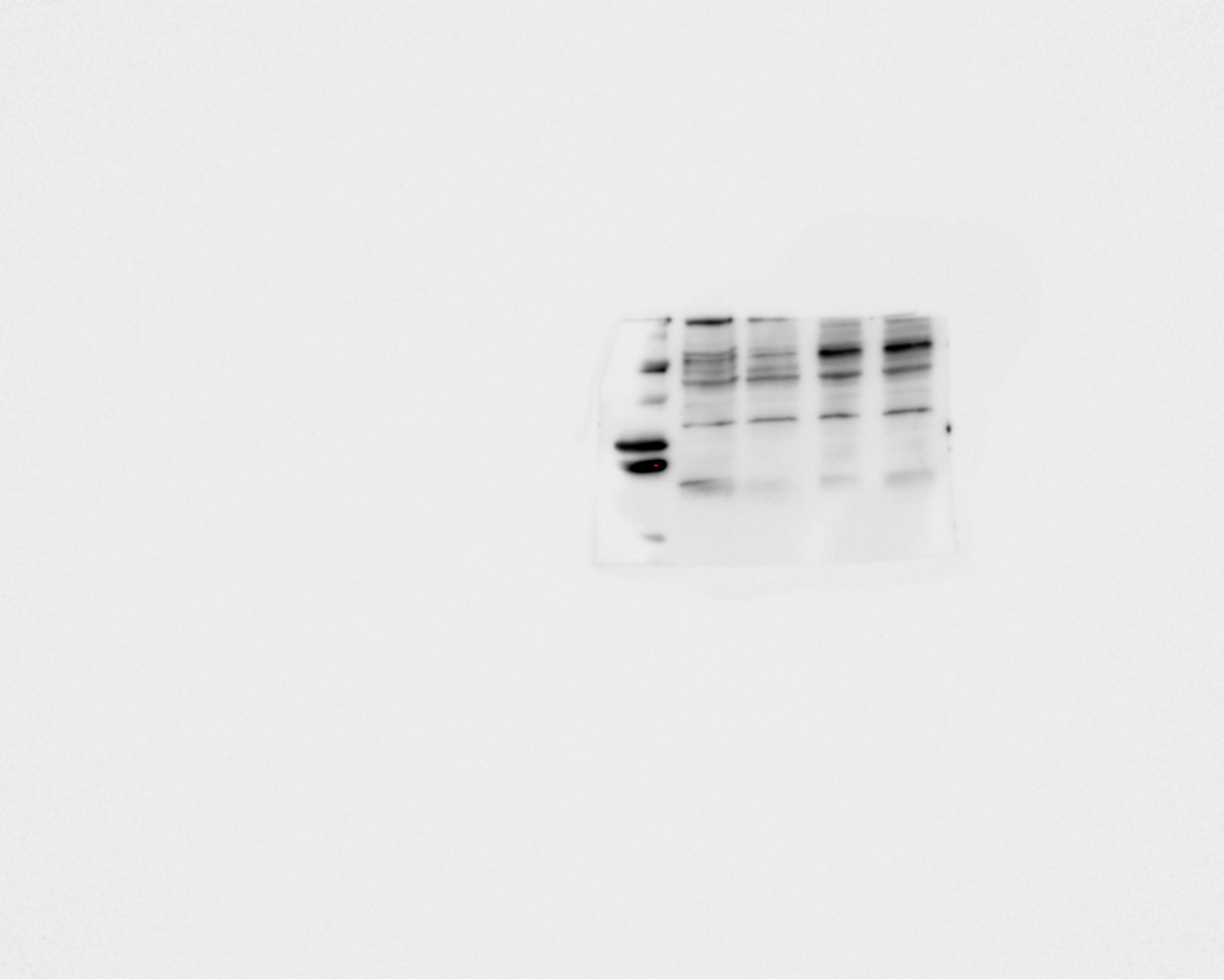
**

**VEGF without ladder**

**
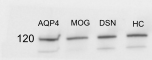
**

**Vinculin**

**IL6 Set 1 and Set 2 with Vinculin**

**
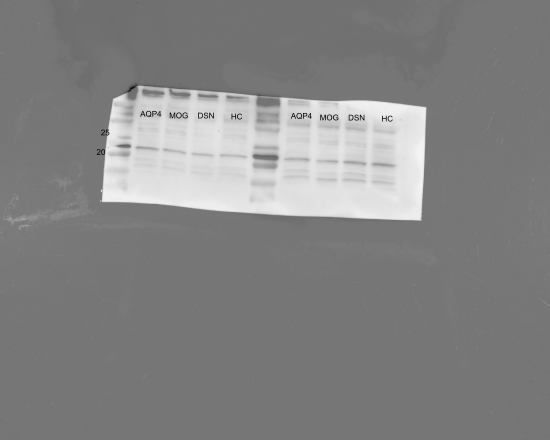
**

**IL6 Set 1 (Left) and Set 2 (Right) with Ladder**

**
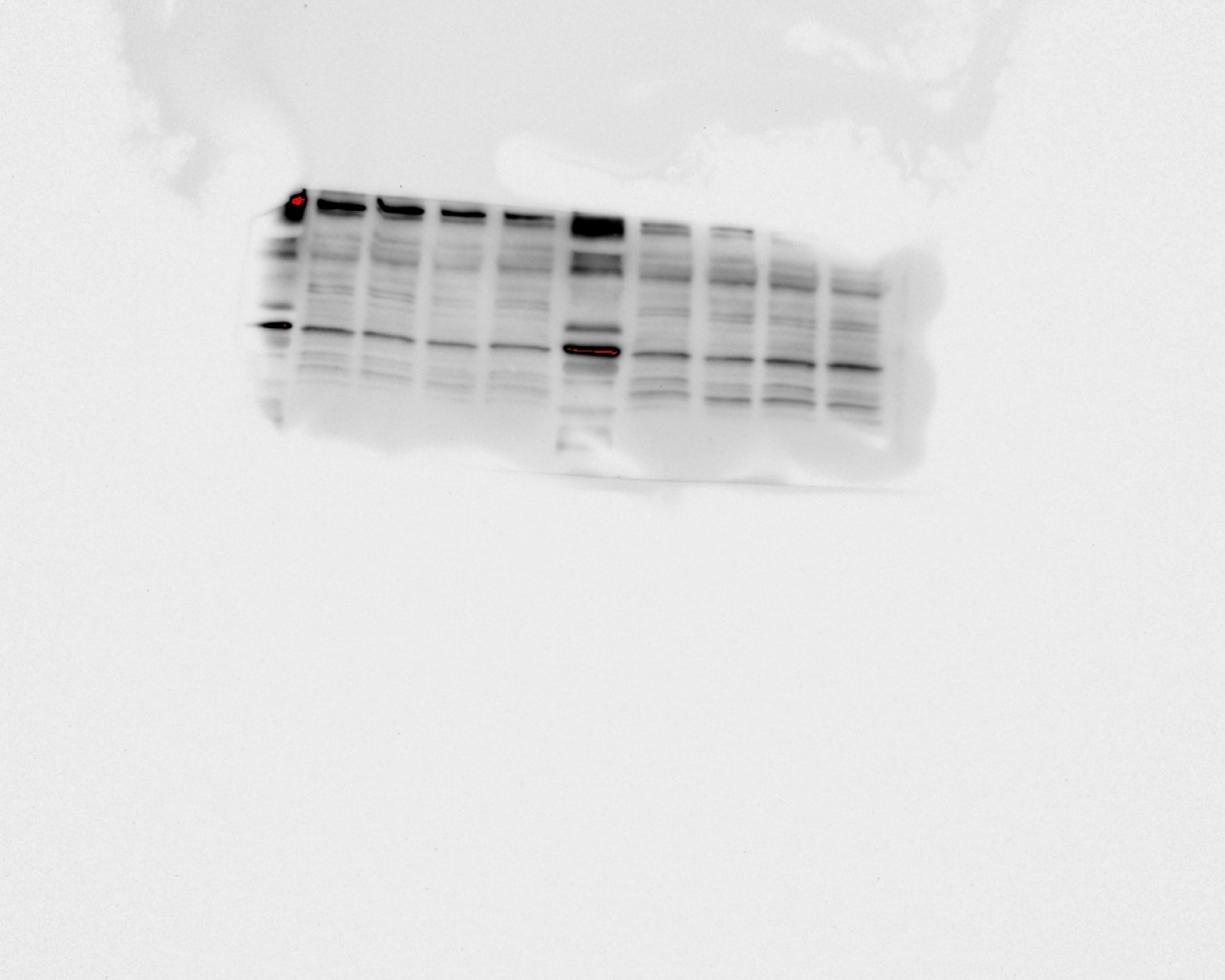
**

**IL6 Set 1 (Left) and Set 2 (Right) without Ladder**

**
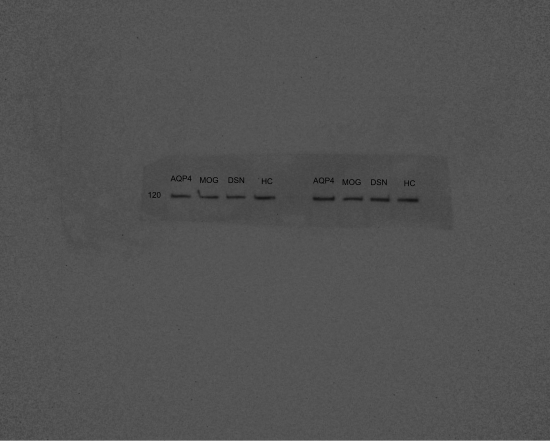
**

**Vinculin with Ladder**

**IL6 Set 3 with Vinculin**

**
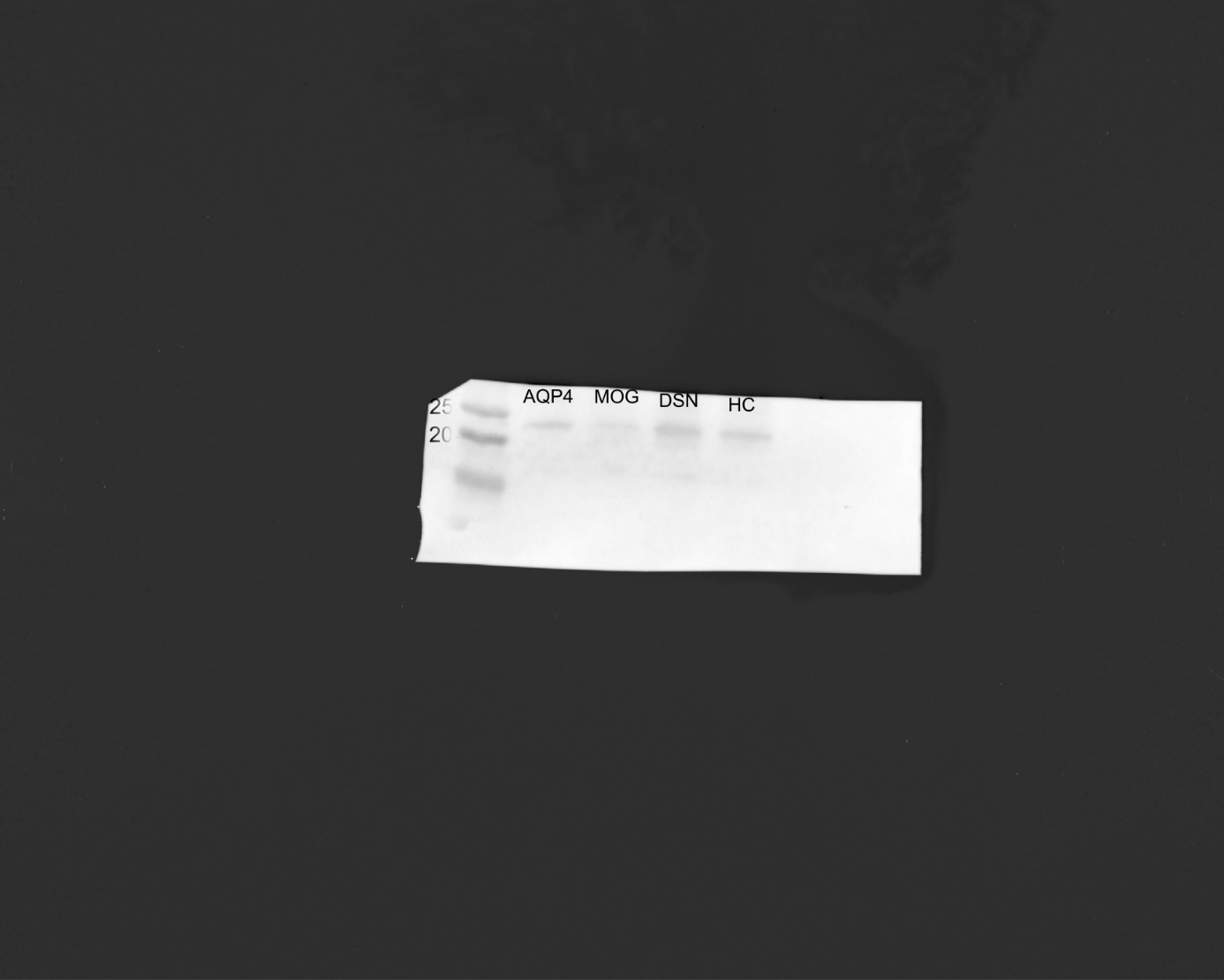
**

**IL6 Set 3 with Ladder**

**
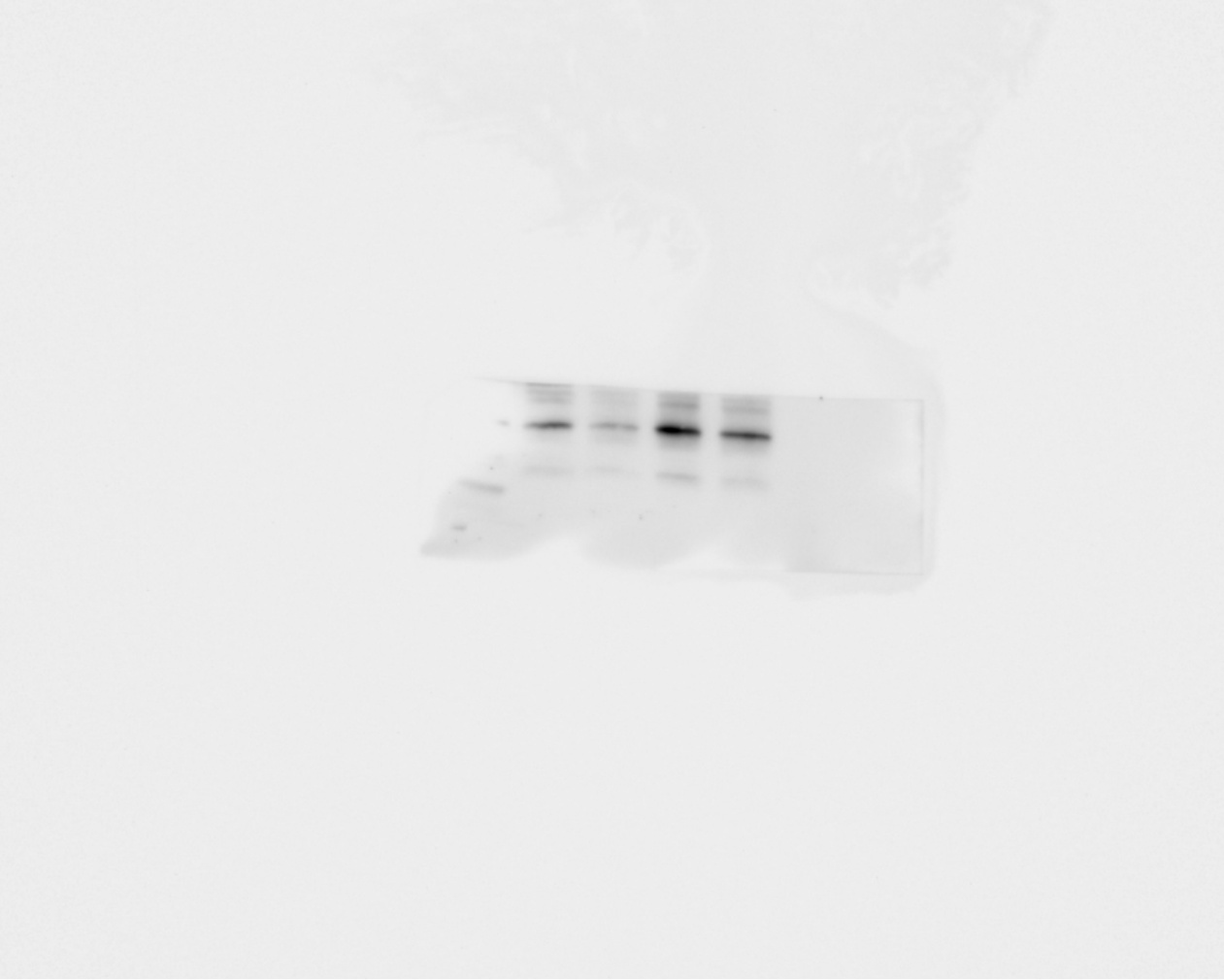
**

**IL6 Set 3 without Ladder**

**
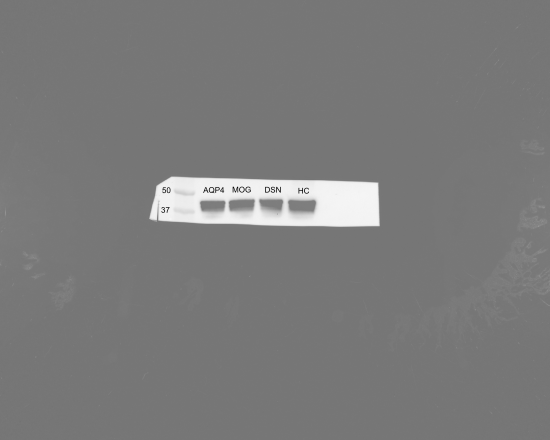
**

**B-actin with ladder**
